# *Brucella* Modulates Secretory Trafficking via Multiple Type IV Secretion Effector Proteins

**DOI:** 10.1371/journal.ppat.1003556

**Published:** 2013-08-08

**Authors:** Sebenzile Myeni, Robert Child, Tony W. Ng, John J. Kupko, Tara D. Wehrly, Stephen F. Porcella, Leigh A. Knodler, Jean Celli

**Affiliations:** 1 Laboratory of Intracellular Parasites, Rocky Mountain Laboratories, National Institute of Allergy and Infectious Diseases, National Institutes of Health, Hamilton, Montana, United States of America; 2 Genomics Unit, Research Technologies Section, Rocky Mountain Laboratories, National Institute of Allergy and Infectious Diseases, National Institutes of Health, Hamilton, Montana, United States of America; 3 Paul G. Allen School for Global Animal Health, College of Veterinary Medicine, Washington State University, Pullman, Washington, United States of America; Duke University, United States of America

## Abstract

The intracellular pathogenic bacterium *Brucella* generates a replicative vacuole (rBCV) derived from the endoplasmic reticulum via subversion of the host cell secretory pathway. rBCV biogenesis requires the expression of the Type IV secretion system (T4SS) VirB, which is thought to translocate effector proteins that modulate membrane trafficking along the endocytic and secretory pathways. To date, only a few T4SS substrates have been identified, whose molecular functions remain unknown. Here, we used an *in silico* screen to identify putative T4SS effector candidate proteins using criteria such as limited homology in other bacterial genera, the presence of features similar to known VirB T4SS effectors, GC content and presence of eukaryotic-like motifs. Using β-lactamase and CyaA adenylate cyclase reporter assays, we identified eleven proteins translocated into host cells by *Brucella*, five in a VirB T4SS-dependent manner, namely BAB1_0678 (BspA), BAB1_0712 (BspB), BAB1_0847 (BspC), BAB1_1671 (BspE) and BAB1_1948 (BspF). A subset of the translocated proteins targeted secretory pathway compartments when ectopically expressed in HeLa cells, and the VirB effectors BspA, BspB and BspF inhibited protein secretion. *Brucella* infection also impaired host protein secretion in a process requiring BspA, BspB and BspF. Single or combined deletions of *bspA*, *bspB* and *bspF* affected *Brucella* ability to replicate in macrophages and persist in the liver of infected mice. Taken together, these findings demonstrate that *Brucella* modulates secretory trafficking via multiple T4SS effector proteins that likely act coordinately to promote *Brucella* pathogenesis.

## Author Summary

Many intracellular parasites ensure their survival and proliferation within host cells by secreting an array of effector molecules that modulate various cellular functions. Among these, *Brucella abortus*, the causative agent of the worldwide zoonosis brucellosis, controls the intracellular trafficking of its vacuole, the *Brucella*-containing vacuole (BCV), towards compartments of the secretory pathway via the expression of a Type IV secretion system (T4SS), VirB, which is thought to translocate effector proteins. Here, we have used bioinformatic algorithms and protein translocation reporter assays to identify novel *Brucella* proteins translocated into host cells, some of which are VirB T4SS substrates and targeted secretory pathway compartments when ectopically expressed in mammalian cells. Three VirB effectors, BspA, BspB and BspF, inhibited protein secretion and contributed to varying degrees to bacterial inhibition of host protein secretion, pathogen intracellular growth and persistence in the liver of infected mice. These findings demonstrate that *Brucella* modulates secretory trafficking via multiple T4SS effector proteins to promote *Brucella* pathogenesis.

## Introduction

Intracellular parasites have evolved specialized mechanisms that exploit a variety of host cellular pathways to generate idiosyncratic niches of replication or persistence. Among these, various bacterial pathogens including *Brucella* spp., *Legionella pneumophila*, *Chlamydia* spp. *and Salmonella enterica* serovar Typhimurium target several compartments of the secretory pathway to promote their replication [1]. The secretory pathway orchestrates the synthesis, modification and transport of proteins and lipids [2]. It is organized into successive membrane-bound compartments including the endoplasmic reticulum (ER), ER-to-Golgi intermediate compartment (ERGIC), Golgi apparatus, trans-Golgi network (TGN), and the plasma membrane [3]. Secretory cargo is selected and transported from ER exit sites (ERES) to the Golgi apparatus via the sequential action of COPII and COPI coat complexes, the activities of which are regulated by Rab-family and ARF-family small GTPases [4], [5], which are targets of bacterial modulation [1].

*Brucella* spp. are Gram-negative intracellular pathogens of various mammals that cause the worldwide zoonotic disease known as brucellosis or Malta fever [6]. Key to the pathogenesis of these bacteria is their ability to infect both phagocytic and non-phagocytic cells ranging from macrophages and dendritic cells to epithelial cells [7], [8], [9], [10]. Upon entry into host cells, *Brucella* reside within a membrane-bound compartment known as the *Brucella*-containing vacuole (BCV), whose trafficking along the endocytic and secretory pathways is controlled by the bacterium [11], [12], [13]. During maturation along the endocytic pathway, the BCV is acidified and acquires late endosomal markers such as Rab7 and LAMP1 [8], [9], [13], before being redirected towards the early secretory pathway through intimate interactions with ERES, eventually fusing with the ER in a process that depends upon the small GTPase Sar1 and thus on the formation of COPII-dependent transport vesicles [14]. During this process, the BCV recruits the small GTPase Rab2 and GAPDH [15] that regulate membrane traffic between the ER and ERGIC, and are required for *Brucella* replication.

Biogenesis of the rBCV depends upon the VirB Type IV secretion apparatus [8], [13], [16], [17], [18], a crucial virulence factor of *Brucella* that delivers effector molecules into the host cell [19], [20] that are thought to modulate BCV trafficking. Importantly, the VirB Type IV secretion apparatus is essential for *Brucella* pathogenesis, since *virB* mutants are incapable of survival and replication in host cells and attenuated in a mouse model of infection [8], [14], [16], [17], [18]. Recently, a number of *Brucella* VirB-dependent effector proteins have been identified [19], [20]. The first VirB substrates, VceA and VceC, were uncovered by screening for genes co-regulated with the *virB* operon [19], of which VceC induces inflammation through the induction of ER stress [21]. Since then, various strategies have been implemented to identify *Brucella* effectors, including the use of *in silico* screening for proteins with distinct features [20], a strategy that has proven to be successful in identifying T4SS effectors of other intracellular pathogens such as *L. pneumophila* and *Coxiella burnetti* [22], [23]. Using this approach, four proteins, BPE123, BPE043, BPE005 and BPE275, were identified as VirB substrates [20], yet their molecular functions and roles in *Brucella* pathogenesis remain unknown. A high throughput yeast two-hybrid screening approach for potential host interactors also recently identified RicA, a protein translocated in a VirB-dependent manner that interacts with Rab2 [24].

Despite our understanding of the VirB T4SS roles in the *Brucella* intracellular cycle and the identification of several effector proteins, VirB-associated molecular functions and the cellular pathways that *Brucella* effectors modulate to control the bacterium's intracellular trafficking still remain unknown. In particular, given *Brucella's* reliance on cellular processes associated with the secretory pathway, determining how the VirB T4SS-mediated functions interfere with this compartment is key to a molecular understanding of *Brucella* pathogenesis. In an effort to identify novel *Brucella* VirB T4SS effector proteins, here we have used a genome-wide *in silico* analysis and protein translocation reporter assays. We report the identification of 11 *Brucella* proteins translocated into host cells, among which 10 are novel and at least 5 are translocated in a VirB T4SS-dependent manner. Importantly, several of these proteins target compartments of the secretory pathway and contribute to *Brucella* interference with host cellular protein secretion, intracellular proliferation and persistence *in vivo*. This study demonstrates *Brucella* modulation of host secretion via novel effector proteins and provides the first evidence of a role of VirB T4SS effector proteins on intracellular membrane trafficking important to *Brucella* pathogenesis.

## Results

### *In silico* identification of putative *Brucella* VirB T4SS effector proteins

To identify *Brucella* putative VirB T4SS effector proteins, we chose to focus on *Brucella* predicted proteins annotated as hypothetical proteins of unknown functions, based on the assumption that such effectors fulfill functions specific to *Brucella* intracellular pathogenesis and should consequently be either restricted to the *Brucella* genus or conserved mostly among closely related α2- proteobacteria. Additionally, we reasoned that they may possess a positive net charge in their C-terminal end and possibly Arginine-rich motifs, which are features of known VirB effector proteins in the closely related organisms *Agrobacterium tumefaciens* and *Bartonella henselae* [25], [26]. Out of the 3,085 predicted proteins from the *Brucella abortus* strain 9-941 genome sequence annotated in the ERGO Genome Analysis Suite (http://integratedgenomics.com/ergo.html), 823 proteins without assigned functions were examined using the Blastp algorithm (http://blast.ncbi.nlm.nih.gov/Blast.cgi) for limited similarities with proteins in other bacteria, and the net charge of their C-terminal 20 amino-acids residues calculated using an EMBOSS bioinformatics tool called “charge” (http://emboss.bioinformatics.nl/). Using this analysis, 93 proteins that were either *Brucella*-specific or highly represented within the α2- proteobacteria and contained a positively-charged C-terminus were selected for further screening (Table S1). An additional criterion for selection was the deviation in GC content from that of the entire genome (Table S1), based on the possibility that VirB T4SS effectors may have been acquired by horizontal transfer. Further analyses used an array of bioinformatics tools including SMART (http://smart.embl-heidelberg.de/), TMpred (http://www.ch.embnet.org/software/TMPRED_form.html), Coils (http://www.ch.embnet.org/software/COILS_form.html), and SignalP v4.1 (http://www.cbs.dtu.dk/services/SignalP/) for the presence of functional or structural domains and presence or absence of Sec-dependent secretion signals, following the assumption that VirB T4SS effector proteins may possess domains or motifs either present in eukaryotic proteins or consistent with interactions with, and modulation of host factors. This combinatorial analysis led to the selection of 20 predicted proteins as candidate VirB T4SS substrates that fulfilled most or parts of the selection criteria (Table S2). Additionally, a predicted protein encoded by the locus BAB1_1671 was independently selected based on the presence of a coiled-coil domain and a C-terminal transmembrane domain in an arrangement reminiscent of eukaryotic membrane fusion soluble NSF attachment protein receptor (SNARE) proteins (Table S2).

### Translocation of *Brucella* putative effector proteins

**Figure 1:**
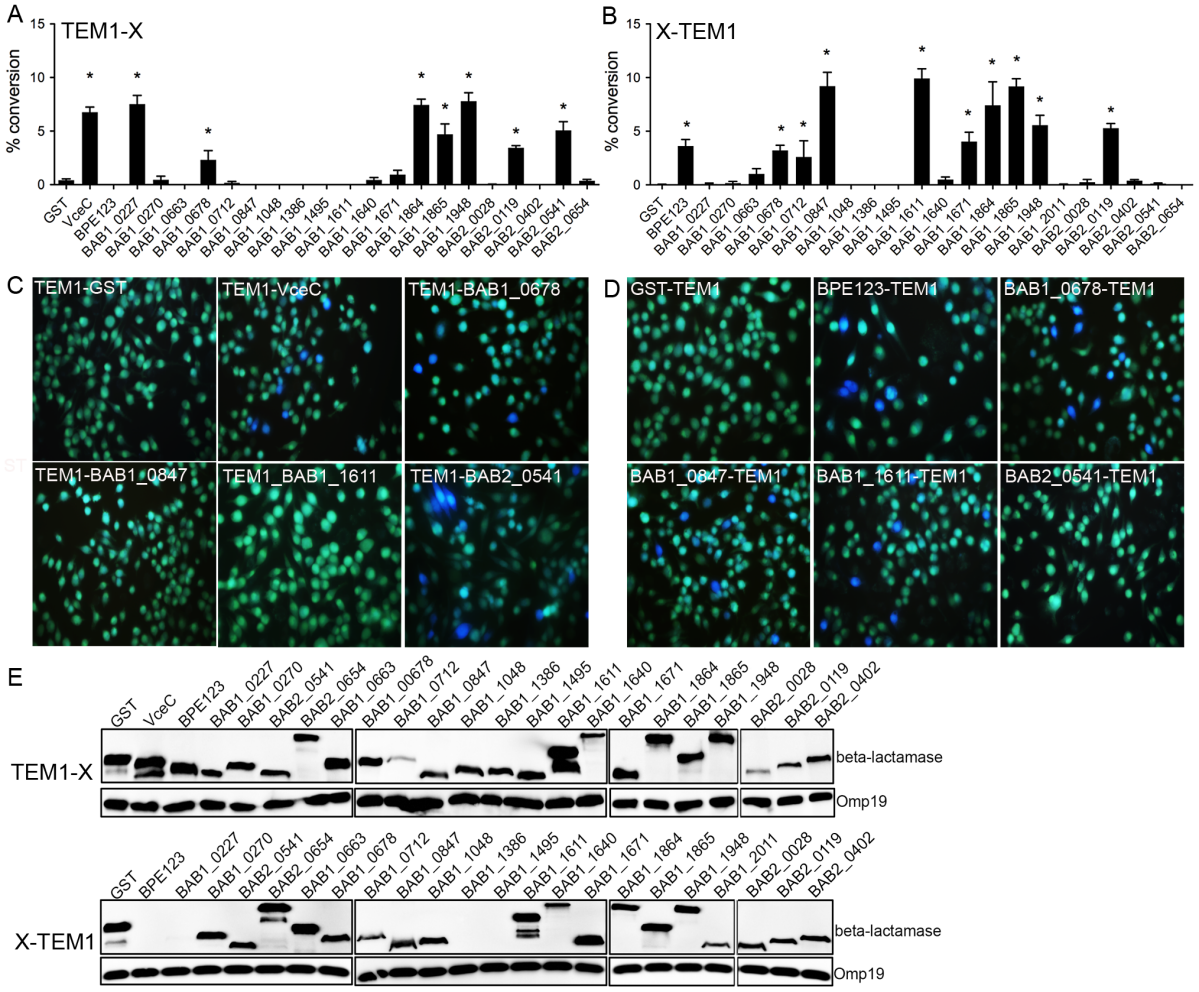
Translocation of *Brucella* putative effector proteins into J774A.1 cells. (A and B) Quantification of the translocation of N-terminally- (A) or C-terminally- (B) TEM1-tagged *Brucella* proteins. The cytosolic translocation of β-lactamase by *B. abortus* strain 2308 expressing the different TEM1 fusion proteins was assessed by fluorescence microscopy in J774.A1 macrophages after 16 h of infection. Cells from ten independent random fields were counted and the percentage of blue cells calculated. Results shown are the mean ± SD of three independent experiments. TEM1-GST and GST-TEM1 were used as negative controls (green fluorescence) and TEM1-VceC as a positive control (blue fluorescence). (C and D) Representative fluorescence micrographs from individual assay wells showing control proteins (GST and VceC) and selected TEM1 fusion proteins tagged at either the N-terminus (C) or C-terminus (D). (E) Western blot analysis showing expression of TEM-1 fusions. Bacterial lysates from *B. abortus* 2308 strain expressing TEM-1 fusion proteins were resolved using SDS-PAGE and subjected to Western blot analysis with anti- β-lactamase TEM-1 antibody. Detection of *Brucella* Omp19 was used as a loading control.

To test whether the *Brucella* putative VirB T4SS effector proteins are translocated into host cells during infection, we first used the TEM1 β-lactamase protein translocation reporter assay [27] previously employed to identify *Brucella* VirB T4SS substrates [19], [24]. Based on a shift in fluorescence emission by the membrane-permeant β-lactamase substrate CCF2/AM upon β-lactamase translocation into host cells, this assay allows for the detection and quantification of protein translocation in cells infected with *Brucella* strains expressing TEM1 fusions with putative translocated effectors [27]. Given the lack of knowledge of VirB-mediated translocation signals in *Brucella*, translational fusions of the TEM1 protein with either the N-terminus or the C-terminus of each putative effector protein were generated and expressed under the control of the isopropyl-ß-D-thiogalactopyranoside (IPTG)-inducible *Ptrc* promoter in either pJC120 or pJC121, respectively (Table S2), in wild-type *B. abortus* 2308. With the exception of BAB1_0227-TEM1, BAB1_1386-TEM1 and BAB1_1495-TEM1, the expression of all fusions was detected by Western blot analysis using anti-β-lactamase and anti-*Brucella* outer membrane lipoprotein Omp19 antibody as loading control, albeit to varying levels (Fig. 1E).

To test translocation of these TEM1 fusions, J774.A1 macrophage-like cells were infected with TEM1 fusion-expressing wild type *Brucella* strains at an MOI of 1000 for 16 h and processed for fluorescence microscopy analysis. TEM1-GST and GST-TEM1 fusion proteins were used as negative controls of translocation and a TEM1-VceC fusion [19] and TEM1 fusions to the BPE123 effector protein [20] were used as positive controls. Expression of the TEM1-VceC fusion triggered CCF2/AM conversion in infected J774.A1 cells, indicating protein translocation, while almost all cells infected with *Brucella* strains expressing either TEM1-GST or GST-TEM1 fusions did not show any translocation (Fig. 1A–D). Additionally, translocation of BPE123-TEM1 was observed (Fig. 1A–B and D), despite undetectable expression (Fig. 1E), but not that of TEM1-BPE123, consistent with the previous demonstration of this effector's translocation [20]. Under these validated experimental conditions, 11 of the 21 *Brucella* putative effector proteins were repeatedly translocated into host cells (Fig. 1, S1 and S2). Ten of these *Brucella* proteins, BAB1_0227, BAB1_0678, BAB1_0712, BAB1_0847, BAB1_1611, BAB1_1671, BAB1_1864, BAB1_1948, BAB2_0119 and BAB2_0541 were newly identified as translocated by *Brucella* into host cells, while translocation of BAB1_1865 (also named BPE865) was previously reported [20]. BAB1_0678, BAB1_1864, BAB1_1865, BAB1_1948 and BAB2_0119 were translocated into host cells regardless of the N- or C-terminal tagging with TEM1 (Fig. 1A–D). However, only the N-terminal TEM1 fusions of BAB2_0541 and BAB1_0227 showed translocation (Fig. 1A–D). While BAB2_0541 fusion constructs were expressed to comparable levels, the BAB1_0227-TEM1 fusion protein was not detected by Western blot analysis (Fig. 1E), potentially explaining its lack of translocation. Yet, the inability to detect protein expression in *Brucella* did not hinder the translocation of BAB2_0123-TEM1 fusion (Fig. 1B, D and E), suggesting that very low levels are sufficient to detect translocation. Conversely, only the C-terminal TEM1 fusions to BAB1_0712, BAB1_0847, BAB1_1611 and BAB1_1671 were translocated, even-though both N- and C-terminal constructs were expressed in *Brucella* (Fig. 1E). Taken together, these results demonstrate that the majority (11 out of 21) of our putative *Brucella* effector proteins identified *in silico* are translocated by *Brucella* during infection and the position of the TEM1 tag at either the N- or C-terminus of the protein can influence its translocation in this assay.

### VirB T4SS-dependent translocation of *Brucella* effectors

To determine whether the newly identified *Brucella* effector proteins were translocated in a VirB T4SS-dependent manner, we needed to examine their translocation by a VirB-deficient strain. Given that *virB* mutants do not survive in macrophages and are progressively killed [8], [14], a translocation assay in which similar numbers of intracellular viable wild type and VirB-deficient bacteria are present was required to generate conclusive data. Consistent with previous reports [8], [14], [16], infection of J774A.1 cells with both the wild type 2308 strain and an isogenic Δ*virB9* mutant showed a progressive decrease in recovery of viable Δ*virB9* bacteria, reaching a 2 Log difference with 2308 by 16 h pi (Fig. S3A), a time point required by our β-lactamase reporter system. We therefore chose to use the *B. pertussis* calmodulin-dependent adenylate cyclase (CyaA) reporter system [28], which has previously identified substrates of the VirB T4SS of *Brucella* [20], at an infection time point (6 h pi) where MOI adjustments yielded similar numbers of recoverable CFUs for both wild type and VirB-deficient bacteria (Fig. S3B).

**Figure 2:**
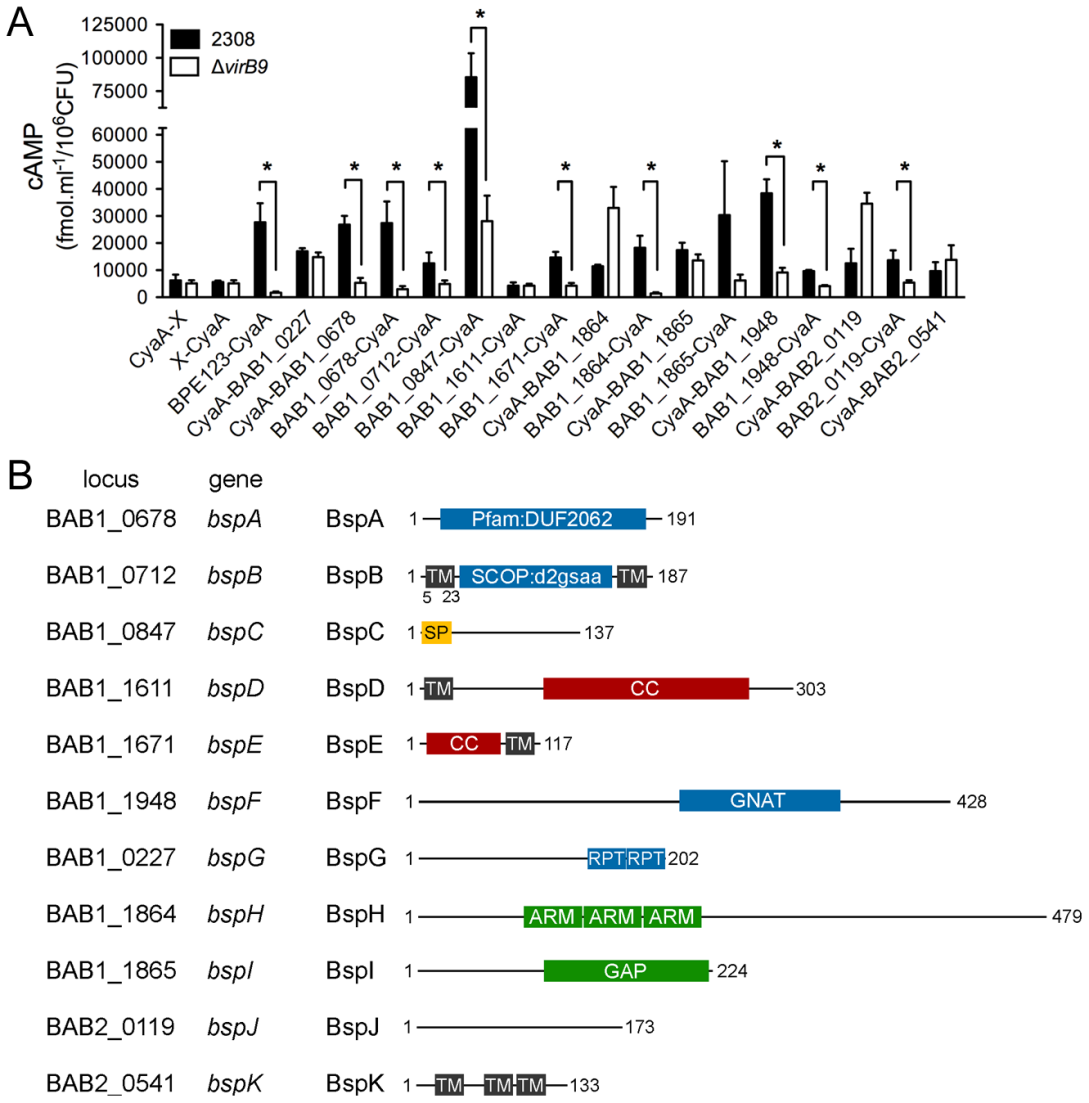
VirB-dependent translocation of *Brucella* Bsp effectors into J774.A1 cells. (A) *B. abortus* strain 2308 (black bars) or the Δ*virB9* mutant strain (white bars) expressing the CyaA fusions to the indicated *Brucella* proteins were used to infect J774.A1 cells, and the cAMP level of infected cells was determined. A previously identified VirB effector, BPE123, was used as a positive control while vectors expressing the CyaA domain alone (CyaA-X or X-CyaA) were used as a negative control. Total cAMP levels resulting from translocation of protein fusions were quantified and are expressed as fmol.ml^−1^/10^6^ CFUs. Results are means ± SD from a representative experiment performed in triplicate. (B) Schematic representation of *Brucella* secreted proteins (Bsp proteins), indicating the corresponding locus, protein lengths and predicted domains or motifs.

**Figure 3:**
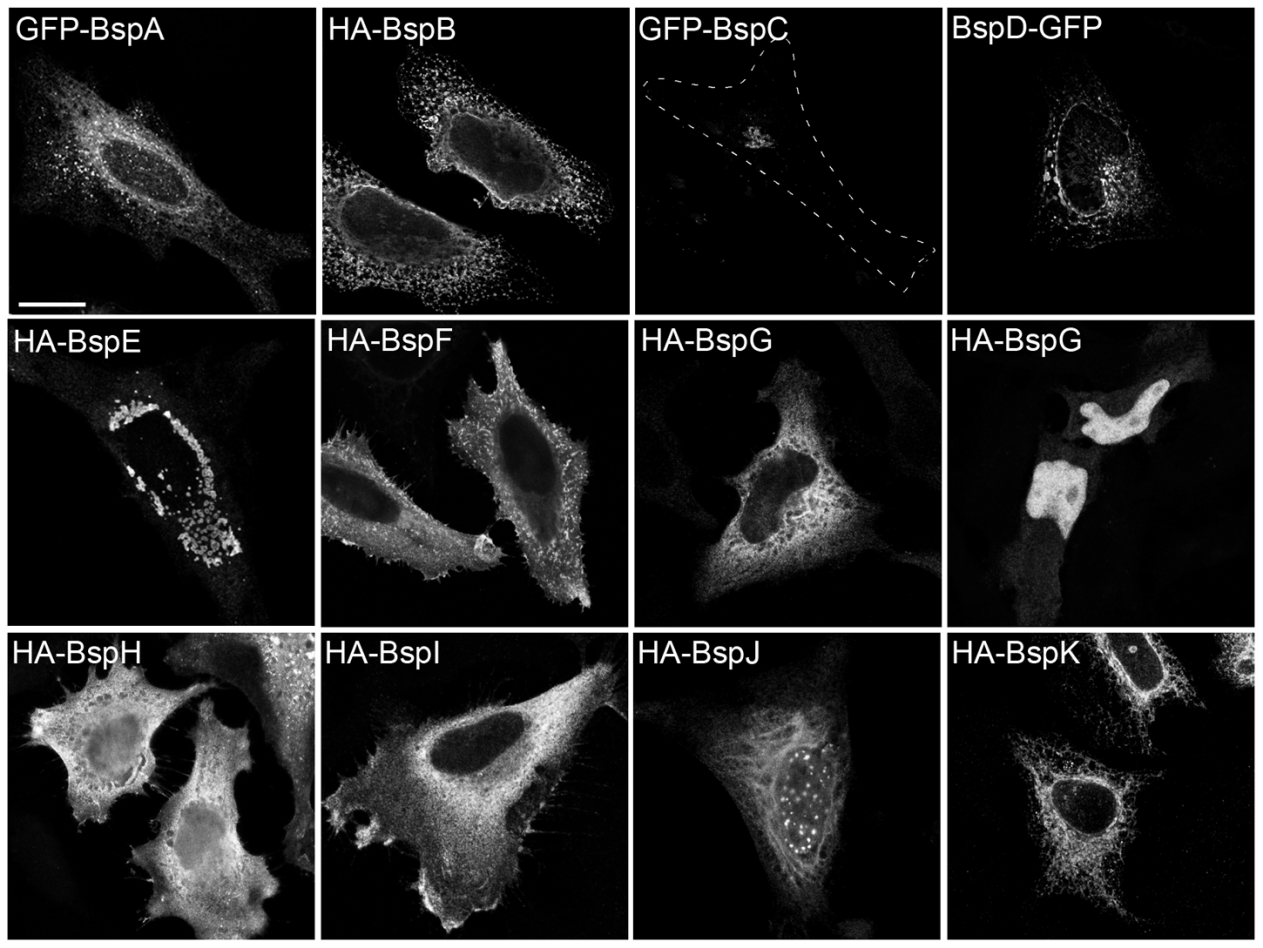
Expression patterns of *Brucella* Bsp effector proteins in HeLa cells. Representative confocal micrographs of HeLa cells transfected for 12 h with plasmids expressing either GFP- or HA-tagged Bsp proteins. HeLa cells expressing HA-tagged *Brucella* effectors were immunostained using an anti-HA antibody. The dashed line on the GFP-BspC panel delineates the cell contour. Scale bar, 10 µm.

CyaA translational fusions with the identified proteins translocated by *Brucella* (Fig. 1A–B) were generated in plasmids pJC125 and pJC126, by positioning the CyaA tags as the TEM1 moieties (Table S2), introduced into either wild type 2308 or the Δ*virB9* mutant strain and their expression was verified by Western blot analysis using a anti-CyaA antibody (data not shown). Cyclic AMP (cAMP) levels were measured in J774.A1 cells infected for 6 h with either wild type or Δ*virB9* strains expressing the CyaA fusions and were normalized to intracellular CFUs to account for variations in infection levels and viability between strains. Compared to pJC125 and pJC126 empty vector controls, expression of the VirB-dependent effector protein BPE123 [20] fused to CyaA generated significant cAMP production in cells infected with the wild type, but not with the Δ*virB9* mutant (Fig. 2A), validating our experimental conditions. Similarly, 10 of the 11 *Brucella* protein fusions showed a significant increase in cAMP levels compared to negative controls (Fig. 2A), confirming our β-lactamase assay results (Fig. 1A–B). Notably, the translocation of BAB1_0678, BAB1_0712, BAB1_0847, BAB1_1671 and BAB1_1948 was VirB T4SS-dependent, as the cAMP levels were significantly lower (P<0.05) after infection with the Δ*virB9* mutant strain (Fig. 2A). Cells infected with Δ*virB9* mutant strains expressing either BAB1_0227 or BAB2_0541 fusion proteins did not show any significant decrease of cAMP levels compared to the wild type strain (Fig. 3), indicating that translocation of these fusion proteins is not VirB-dependent. Intriguingly, VirB-dependency of translocation of the BAB1_1864, BAB1_1865 and BAB2_0119 fusion proteins was inconclusive since the location of the CyaA tag influenced the results: translocation of these fusion proteins was VirB-dependent when the CyaA tag was C-terminal, but VirB-independent when N-terminal (Fig. 2A). Additionally, VirB-dependent translocation of BAB1_1611 could not be assessed, since the CyaA fusion led to protein instability in *Brucella* (data not shown). Taken together, our results demonstrate that we have identified 11 *Brucella* proteins translocated into host cells during infection, at least 5 of which are *Brucella* VirB T4SS effector proteins. We therefore named these proteins *Brucella* secreted proteins (Bsp) BspA (BAB1_0678), BspB (BAB1_0712), BspC (BAB1_0847), BspD, (BAB1_1611), BspE (BAB1_1671), BspF (BAB1_1948), BspG (BAB1_0227), BspH (BAB1_1864), BspI (BAB1_1865), BspJ (BAB2_0119) and BspK (BAB2_0541), of which BspA, BspB, BspC, BspE and BspF are VirB T4SS substrates (Fig. 2B). Interestingly, BspA contains the Pfam domain DUF2062; BspB, a SCOP structural domain (d2gsaa) present in pyridoxal phosphate (PLP)-dependent transferases flanked by 2 predicted transmembrane (TM) domains; BspD and BspE contain Coiled-coil (CC) and TM domains; BspF, a GNAT-family acetyltransferase domain; BspG, internal repeat sequences (RPT) in its C-terminal end; BspH, Armadillo (ARM) repeats; BspI, a GTPase Activating Protein (GAP) domain. BspC is the only translocated protein containing a predicted Sec-dependent signal peptide (Fig. 2B and Table S1).

### Distinct cellular localization patterns of *Brucella* effector proteins

**Figure 4:**
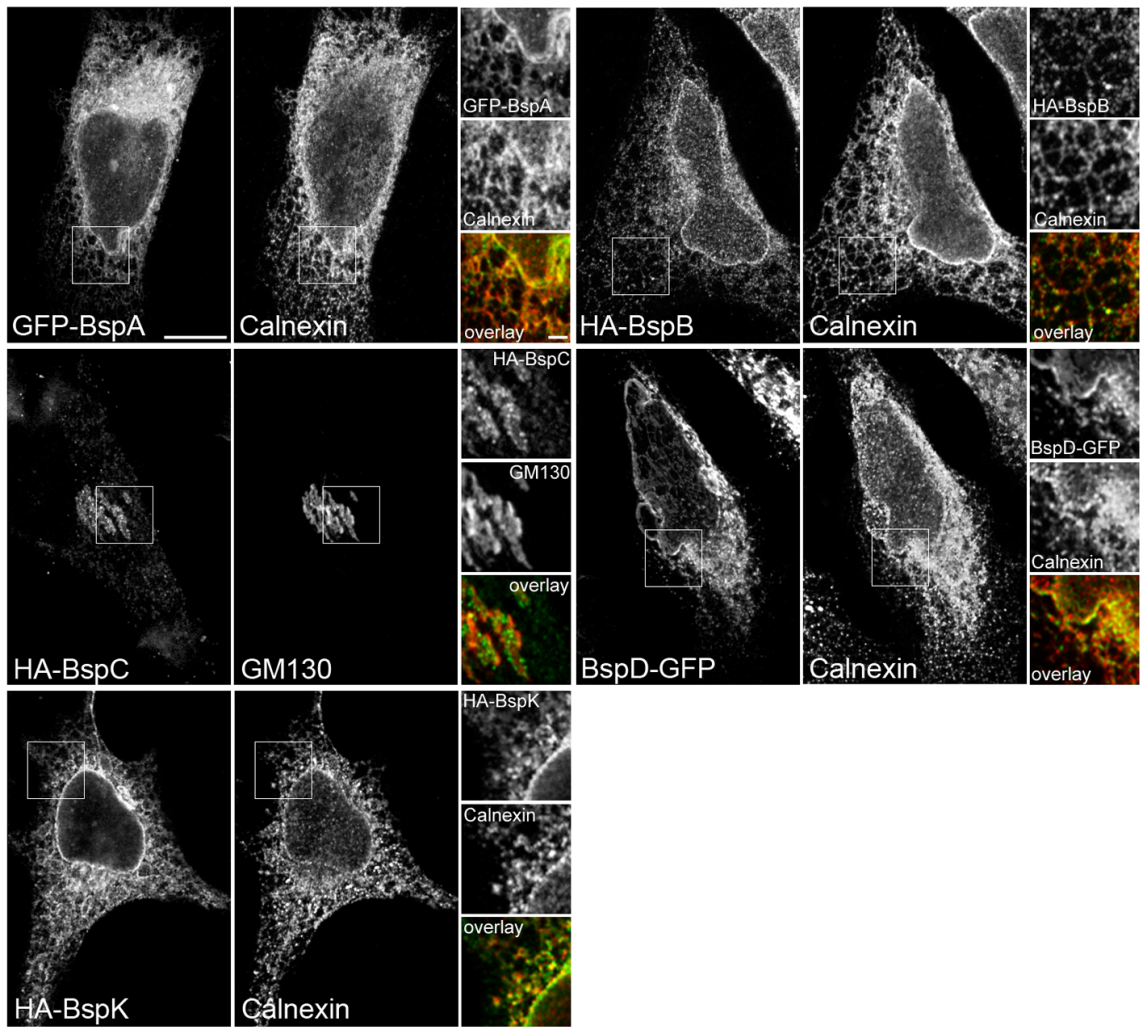
A subset of Bsp effectors target the host secretory pathway. Representative confocal micrographs of HeLa cells transfected with the indicated HA- or GFP-tagged Bsp protein and immunostained for HA (green) and calnexin or GM130 (red) at 12 h post-transfection. Magnified insets show single channel and overlay color images of the boxed region. Scale bar, 10 and 2 µm.

To gain insight into the functions of the Bsp proteins, we first tested whether they target a particular intracellular compartment when expressed ectopically in mammalian cells. HeLa cells were transfected with expression plasmids encoding either Hemagglutinin (HA)- or Green Fluorescent Protein (GFP)-tagged Bsp proteins for 12 h, and processed for fluorescence confocal microscopy analysis for representative expression patterns in low-expressing cells. In most cases, HA- or GFP-tagged Bsp proteins displayed similar expression patterns. While HA-BspH, and HA-BspI displayed a cytosolic localization, HA-BspG partitioned between the cytosol and the nucleus (Fig. 3), with cytosol and nuclear localizations in some cells (data not shown). Similarly, HA-BspJ also partitioned between the cytosol and punctate structures in the nucleus reminiscent of nuclear speckles (Fig. 3). HA-BspE formed discrete vesicles predominantly in the perinuclear area, while HA-BspF localized to both the cytosol and plasma membrane protrusions (Fig. 3). Interestingly, GFP-BspA, HA-BspB, BspD-GFP and HA-BspK displayed distinct reticular expression pattern consistent with a localization to the ER (Fig. 3). Colocalization of GFP-BspA, HA-BspB, BspD-GFP and HA-BspK with the ER-resident chaperone protein, Calnexin (Fig. 4) confirmed that these proteins accumulate in the ER. In addition, GFP-BspC localized to a discrete perinuclear compartment consistent with the Golgi apparatus (Fig. 3), which was confirmed by colocalization of GFP-BspC-positive vesicles with the Golgi marker GM130 (Fig. 4). Hence, ectopically expressed Bsp proteins localize to distinct intracellular compartments in HeLa cells, with 5 out of 11 (BspA, BspB, BspC, BspD and BspK) targeting the secretory pathway.

### BspA, BspB and BspF inhibit host protein secretion

**Figure 5:**
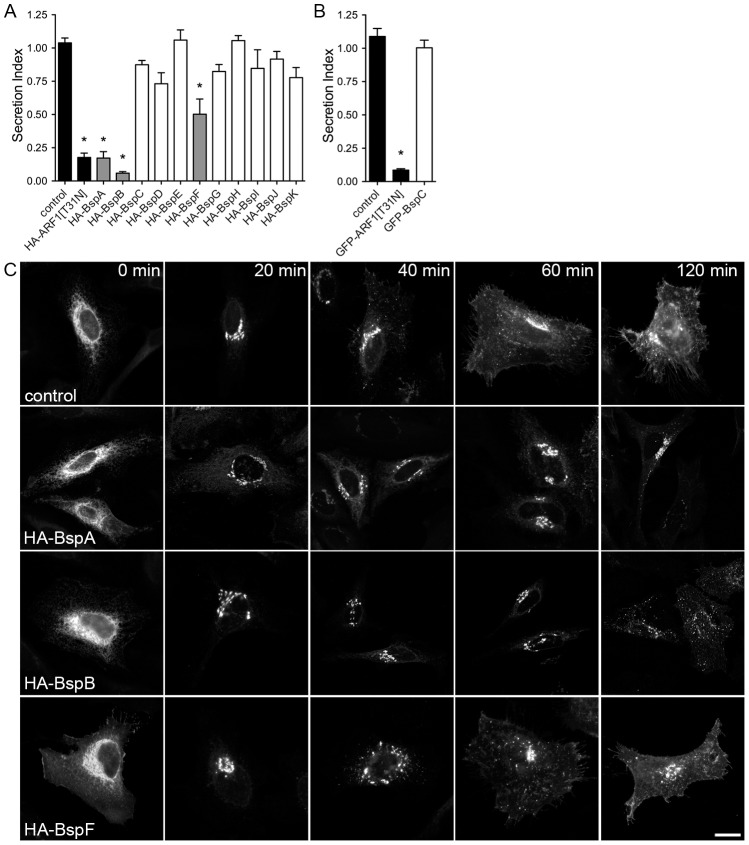
Ectopic expression of BspA, BspB and BspF in HeLa cells interferes with the host secretory pathway. (A and B) Quantitation of SEAP secretion in HEK 293T cells expressing Bsp proteins. HEK 293T cells were co-transfected with plasmid pSEAP-2 and plasmids encoding either HA- (A) or GFP- (B) tagged Bsp proteins are indicated. Twenty-four hours after transfection, SEAP was measured and expressed as a secretion index. Empty vectors served as control and expression of GDP-locked ARF1[T31N] served as a positive control. Data points represent the mean ± SEM of three independent experiments performed in triplicate (^*^ indicates a P value<0.001). (C) Representative confocal micrographs of HeLa cells showing VSV-Gts045-GFP temporal trafficking in HeLa cells expressing the HA-tagged indicated Bsp proteins. HeLa cells were co-transfected with plasmids expressing VSV-Gts045-GFP and the indicated Bsp protein, incubated for 20 h at 40°C. Cells were then treated with cycloheximide and shifted to 32°C at t = 0 min to allow VSV-Gts045-GFP transport to occur. Cells were fixed and immunostained for GFP at the indicated times post-temperature shift. Scale bar, 10 µm.

Given the propensity of the identified Bsp proteins to target the secretory pathway and considering that *Brucella* manipulates functions of this particular compartment to generate the rBCV, we sought to determine whether expression of Bsp proteins affects functions of the secretory pathway. To achieve this, we first examined secretion of a reporter protein, the secreted embryonic alkaline phosphatase (SEAP) [29], in HEK293T cells co-expressing SEAP and HA- or GFP-tagged Bsp proteins. The GDP-locked allele of the small GTPase ARF1[T31N] was used as a control for inhibition of secretion, since its expression disrupts the early secretory pathway [30]. With the exception of HA-BspC, all HA- or GFP-tagged Bsp proteins were expressed in HEK293T cells when analyzed by Western blot using anti-HA or anti-GFP antibodies, although to varying levels (Fig. S4A). After 24 h of co-transfection, HEK293T cells were processed for SEAP secretion analysis and the secretion index calculated as the extracellular SEAP activity versus intracellular SEAP activity normalized to transfections with the SEAP plasmid only. While cotransfections of empty vectors with the SEAP plasmid did not alter the levels of secreted SEAP, expression of HA- or GFP-tagged ARF1[T31N] led to a ∼80% reduction in SEAP secretion (Fig. 5A and B). Interestingly, expression of HA-BspA, HA-BspB, or HA-BspF led to a significant decrease in SEAP secretion, with BspF inhibiting secretion by ∼50%, while BspA and B showed a stronger inhibition in secretion (>80%) to levels comparable to the effect of ARF1[T31N] (Fig. 5A). The levels of secretion inhibition did not correlate with Bsp expression levels, since BspA and BspB showed lower expression than BspF (Fig. S4A). In comparison, expression of either BspC, BspD, BspE, BspG or BspJ did not affect SEAP secretion levels (Fig. 5A–B), regardless of their expression levels (Fig. S4A). Since expression of BspC, BspD or BspK did not alter SEAP secretion despite their targeting of secretory compartments (Fig. 3 and 4) and BspF does not accumulate in a secretory compartment yet impairs SEAP secretion (Fig. 3–5), BspA-, BspB- or BspF-mediated inhibition of SEAP secretion is not simply due to their accumulation along the secretory compartment. Rather, inhibition of protein secretion may result from specific activities of these effectors.

The decrease of processing in the secretory pathway is a hallmark of ER stress [31], which can quickly down regulate SEAP secretion [32]. To examine whether the decrease in SEAP secretion observed upon expression of either BspA, BspB or BspF was due to induction of ER-stress, we monitored this response in cells expressing these proteins using the ERSE reporter (*luc*) dual-luciferase assay and expression of downstream protein targets of ER stress, CHOP and BiP/GRP78 [33]. Tunicamycin, an ER stress stimulus that inhibits NH_2_-linked glycosylation and protein folding in the ER, and expression of HA-VceC, a *Brucella* VirB T4SS effector that induces ER stress and proinflammatory cytokine expression upon ER stress induction [21] were used as positive controls in these assays (Fig. S4B). Compared to Tunicamycin treatment or VceC expression, which induced ER stress to varying degrees, expression of either HA-BspA, HA-BspB or HA-BspF did not induce ER stress (Fig. S4B–C), nor did expression of HA-BspD, HA-BspE, HA-BspH or HA-BspI, although HA-BspF increased BiP levels (Fig. S4C). In comparison, GFP-BspC, HA-BspG or HA-BspK expression significantly induced ER stress to levels similar or higher than VceC and upregulation of BiP, but not CHOP (Fig. S4B–C). These results verify that inhibition of SEAP secretion by BspA and BspB is not due to induction of ER stress, while this remains unclear for BspF due to its ability to increase levels of BiP expression, and also demonstrates that BspC, BspG and BspK induce significant ER stress when ectopically expressed in mammalian cells.

Since inhibition of secretory transport can also result from the physical disruption of secretory compartments [34], [35], [36], we also examined the effect of HA-BspA, HA-BspB or HA-BspF expression on the morphology of the secretory pathway, using immunofluorescence confocal microscopy. In HeLa cells expressing either GFP-BspA, HA-BspB or HA-BspF proteins, no significant difference was observed in the morphology of the ER, ERES, ERGIC or Golgi apparatus (Fig. S5), indicating that the inhibitory effect these proteins have on SEAP secretion does not result from overt physical disruption of the secretory compartment.

To independently confirm that BspA, B and F inhibit host protein secretion, we then examined the anterograde transport of VSV-Gts045, a temperature sensitive mutant reporter protein from the vesicular stomatitis virus that is retained in the ER at a non-permissive temperature of 40°C due to a temperature sensitive misfolding, but can be folded normally and transported to the Golgi and the plasma membrane (PM) at 32°C [37], [38]. HeLa cells were co-transfected with plasmids expressing VSVGts045-GFP and either HA-BspA, HA-BspB or HA- BspF for 20 h at 40°C and then shifted to 32°C to monitor VSV-Gts045-GFP trafficking by fluorescence microscopy. In control cells expressing VSVGts045-GFP alone, the majority of the protein was transported from the ER to the Golgi apparatus within 20 min after temperature shift, and was located at the PM by 60 and 120 min (Fig. 5C). VSVGts045-GFP was transported with apparent similar kinetics in cells expressing BspF (Fig. 5C), but not in cells expressing BspA, where it was strongly retained in the ERGIC and Golgi apparatus even after 120 min (Fig. 5C). Expression of BspB also altered VSVGts045-GFP transport specifically from the Golgi to the PM, since the majority of the mutant protein remained in Golgi compartments for up to 60 min and did not reach the PM by 120 min (Fig. 5C). Hence, BspA and BspB expression in HeLa cells impairs secretory trafficking, consistent with their inhibitory effects on protein secretion (Fig. 5A).

### *Brucella* impairs cellular secretion during infection

**Figure 6:**
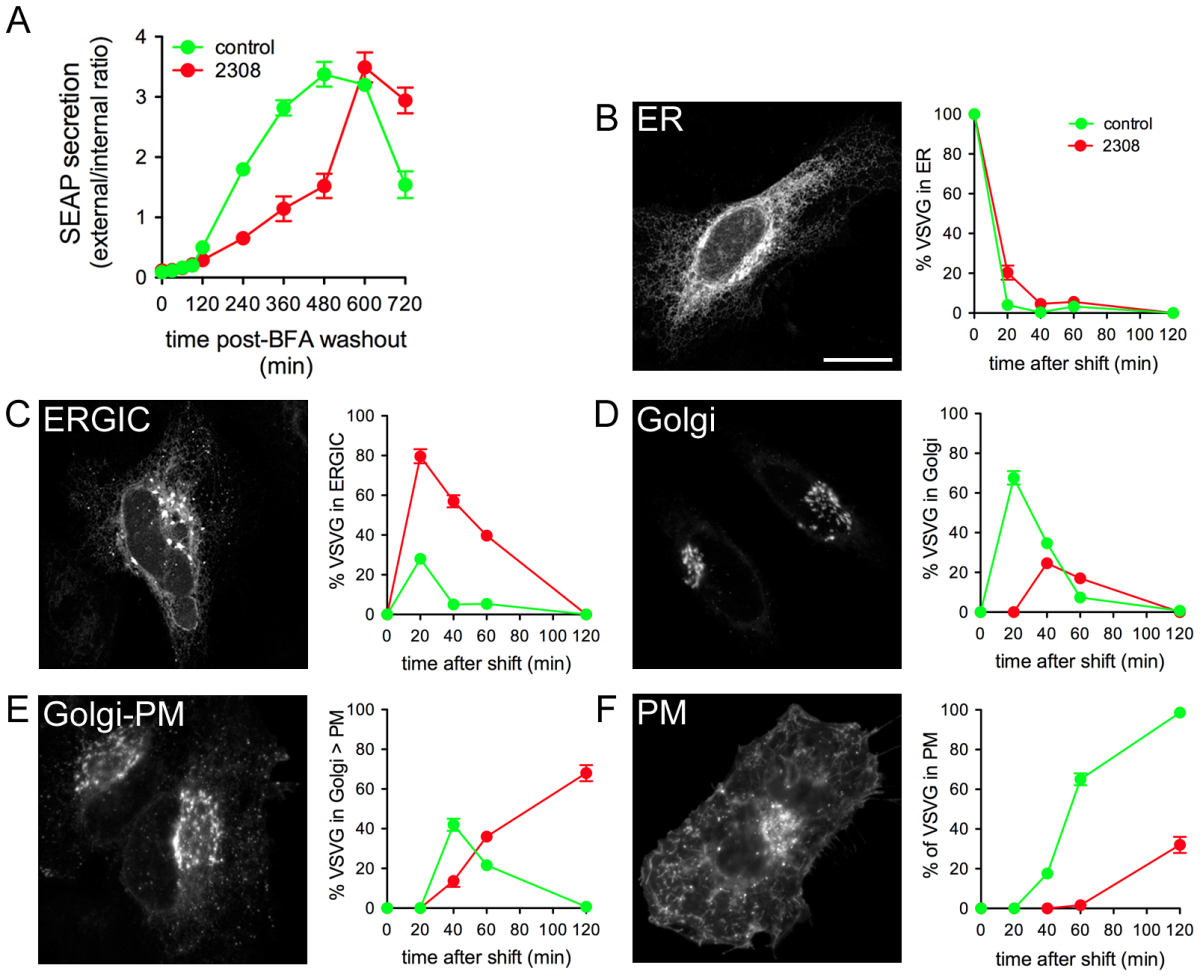
*Brucella* impairs host cell secretion during infection. (A) Quantification of SEAP secretion in uninfected (control) and *B. abortus* 2308-infected (2308) HeLa cells. HeLa cells were transfected with pSEAP-2 for 16 h, then infected with *B. abortus* 2308. At 24 h pi, SEAP protein production was synchronized as described in the Materials and Methods and the external and internal SEAP activity measured at various times post-synchronization. Data show SEAP ratios (external/internal SEAP activity) from uninfected or infected cells. (B–F) VSV-Gts045-GFP trafficking in either control or *B. abortus* 2308-infected HeLa cells. HeLa cells were either mock-infected or infected with DsRed_m_-expressing *B. abortus* 2308. Twenty-four hours pi, cells were transfected with plasmid encoding VSV-Gts045-GFP and incubated at 40°C for 20 h. After 30 min of cycloheximide treatment to stop VSV-Gts045-GFP production, cells were shifted to 32°C, then fixed and immunostained at the indicated time points and VSV-Gts045-GFP localization was examined. (B) Percentage of VSV-Gts045-GFP in the ER, (C) in the ERGIC, (D) in the Golgi apparatus, (E) in the Golgi to plasma membrane (Golgi-PM) compartments and (F) % of VSV-G at the plasma membrane (PM). Data are expressed as a percentage of where VSV-G is located in the defined compartment, as shown in the representative confocal micrographs and are the means ± SD from three independent experiments.

Given the effect of ectopically-expressed BspA, BspB and BspF on host protein secretion, we hypothesized that *Brucella* interferes with secretory transport to the plasma membrane during infection. To test this hypothesis, we monitored host protein secretion during *Brucella* infection utilizing both SEAP and VSV-G transport assays. First, HeLa cells were transfected with the SEAP plasmid for 16 h, then mock-infected or infected with wild type *Brucella* strain 2308. At 24 h pi, SEAP secretion was synchronized by treating cells for 30 min with brefeldin A (BFA) to reversibly disrupt the early secretory pathway and block transport from the ER [39], [40] and cycloheximide to inhibit new SEAP biosynthesis and allow for monitoring secretion of a given pool of SEAP. BFA was then washed out, which allowed Golgi apparatus full reassembly within 2 h [41] with similar kinetics in mock- and *Brucella*-infected cells (Fig. S7), and SEAP secretion was monitored at various time points post washout. Compared to mock-infected cells, *Brucella* infection caused a strong delay in SEAP secretion (Fig. 6A), which was only restored to control levels by 10 h post washout. It is also worth noting that a maxiumum of 30% of cells were both transfected and infected with *Brucella* in these experiments (data not shown), likely causing an underestimation of the inhibitory effect that *Brucella* exerts on cellular secretion. These results demonstrate that *Brucella* trafficking to and replication in the ER affects secretion along the secretory pathway.

*Brucella*-induced delayed transport of a given pool of secretory cargo suggests that this bacterium affects a subcompartment of the secretory pathway that can eventually support trafficking. To investigate further *Brucella* inhibitory effects on secretion, we examined the trafficking kinetics of VSV-Gts045-GFP in *Brucella*-infected cells. HeLa cells were mock-infected or infected with *Brucella* for 24 h and then transfected with VSV-Gts045-GFP for 20 h at 40°C. Confocal micrographs were collected at various time points after a temperature shift to 32°C to quantify the localization of VSV-Gts045-GFP to different subcompartments (ER, ERGIC, Golgi, Golgi to PM and PM) of the secretory pathway. Upon temperature shift, VSV-Gts045-GFP rapidly trafficked out of the ER in mock-infected cells (Fig. 6B) to accumulate in the Golgi apparatus by 20 min (Fig. 6D), undergo post-Golgi trafficking by 40 min (Fig. 6E) and reach the PM by 60 min (Fig. 6F). While VSV-Gts045-GFP trafficking out of the ER was only slightly delayed in infected cells (Fig. 6B), it accumulated and was retained in the ERGIC from 20 to 40 min (Fig. 6C), after which it underwent post-Golgi trafficking (Fig. 6E) but inefficiently reached the PM by 120 min (Fig. 6F). Hence, consistent with the SEAP secretion data (Fig. 6A), these results demonstrate that *Brucella* disrupts host protein secretion by impairing cargo trafficking between the ER and the Golgi apparatus and decreasing transport to the PM.

### BspA, BspB and BspF mediate *Brucella* inhibition of host secretion

**Figure 7:**
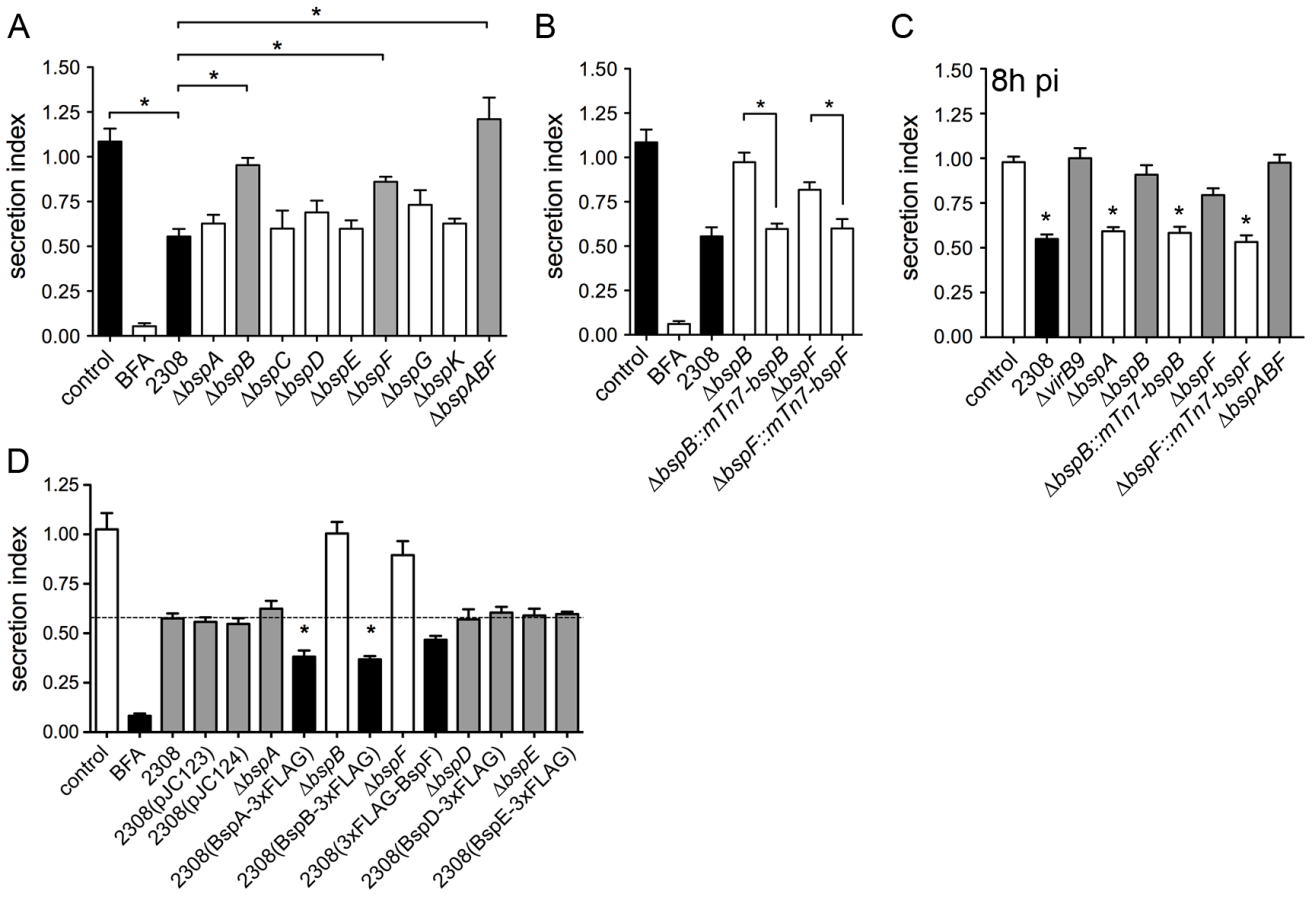
Contribution of BspA, BspB and BspF to inhibition of host cell secretion during infection. (A) SEAP secretion in HeLa cells infected with either wild type or *bsp* in-frame deletion mutants. Brefeldin A (BFA) treatment (5 µg/ml) was used as a control for secretion inhibition. (B) SEAP secretion in HeLa cells infected with either *B. abortus* 2308, Δ*bspB* and Δ*bspF* or the chromosomally-complemented strains Δ*bspB*::*mTn7-bspB* or Δ*bspF*::*mTn7-bspF*. (C) SEAP secretion in HeLa cells infected with either *B. abortus* 2308, Δ*virB9*, Δ*bspA*, Δ*bspB*, Δ*bspF*, the chromosomally-complemented strains Δ*bspB*::*mTn7-bspB* or Δ*bspF*::*mTn7-bspF* or the Δ*bspABF* mutant. (D) SEAP secretion in HeLa cells infected with either *B. abortus* 2308, its isogenic Δ*bspA*, Δ*bspB*, Δ*bspF*, Δ*bspD*, Δ*bspE* mutants, or 2308 carrying pJC123, pJC124 or derivatives expressing FLAG-tagged BspA, BspB, BspF, BspD or BspE. Infection assays were carried out as described in Materials and Methods section and SEAP secretion was measured at 6 h post-synchronization, corresponding to 30 h pi (A, B and D) or 8 h pi (C). Data points represent the means ± SD from three independent experiments performed in quadruplicates. Statistically significant differences (P<0.05) are indicated by asterisks, as compared to control (C) or 2308 (D) samples.

Since *Brucella* disrupts host cell secretion during infection (Fig. 6) and the VirB T4SS effectors BspA, BspB and BspF also affect this process when ectopically expressed (Fig. 5), we next investigated a direct role of these effectors on the host secretory pathway during infection. We therefore generated single or multiple in-frame deletions of *bsp* genes in *B. abortus* strain 2308 (Table S3 and Fig. S6). HeLa cells transfected with the SEAP plasmid were infected with either the wild type 2308 strain or various single *bsp* mutants. At 24 h pi, SEAP secretion was synchronized as described above using BFA and cycloheximide, and measured 6 h post BFA washout, a time point when *Brucella* infection caused significant inhibition of SEAP secretion (Fig. 6A). As a positive control for secretion inhibition, BFA treatment strongly inhibited SEAP secretion when maintained throughout the experiment (Fig. 7A–B). While infection with the wild type 2308 strain significantly reduced SEAP secretion by ∼50%, the Δ*bspB* and Δ*bspF* failed to inhibit SEAP secretion (Fig. 7A–B). Complementation of either *bspB* or *bspF* mutants with a single chromosomal copy of the respective gene restored the inhibitory phenotype (Fig. 7B), demonstrating that BspB and BspF specifically contribute to *Brucella*-mediated inhibition of secretion during infection. By contrast, the Δ*bsp*A mutant strain inhibited SEAP secretion similar to the wild type strain, despite strongly decreasing protein secretion when overexpressed in HEK293T or HeLa cells (Fig. 5), suggesting it does not contribute to inhibition of secretion in the context of an infection. As controls, deletion of either *bspC*, *bspD*, or *bspK*, whose products all target the secretory pathway, did not restore host protein secretion, consistent with their lack of effect on this pathway when ectopically expressed (Fig. 5), and deletion of either *bspE* or *bspG*, which encode two effector proteins unrelated to the secretory pathway (Fig. 3 and 5), did not relieve the secretory inhibition phenotype (Fig. 7A). Taken together, these results demonstrate that BspB and BspF specifically interfere with cellular secretion. Interestingly, the combinatorial deletion of *bspA*, *bspB* and *bspF* rescued the SEAP secretion to levels comparable to uninfected cells expressing SEAP protein alone (control; Fig. 7A) and more completely than upon deletion of single genes, suggesting that at least BspB and BspF coordinately act to impair protein secretion.

Since overexpression of BspA in HeLa cells inhibits host secretion (Fig. 5), yet deletion of *bspA* does not relieve *Brucella*-mediated inhibition of secretion (Fig. 7A), we hypothesized that BspA effect on the host secretory pathway may be counteracted by (an)other unidentified *Brucella* effector(s) that would be absent in an ectopic expression context, and that BspA overexpression in *Brucella* may enhance and reveal its inhibitory effect on secretion. To test this hypothesis, BspA, BspB, BspF, BspD and BspE fused to a 3×FLAG tag according to their translocation requirements (Fig. 1 and 2) were expressed under IPTG-inducible *Ptrc* promoters on multicopy plasmids pJC123 or pJC124 in the wild-type 2308 strain (Fig. S8) and SEAP secretion was measured in infected HeLa cells. Compared to the secretory inhibition caused by the wild-type strain or strains carrying empty vector controls and under experimental conditions where both the Δ*bspB* and Δ*bspF* mutants failed to inhibit protein secretion, overexpression of BspA-3×FLAG and BspB-3×FLAG significantly enhanced inhibition of SEAP secretion, while that of 3×FLAG-BspF did not (Fig. 7D). This increased inhibition of host secretion was specific to these effectors, since it was not observed upon overexpression of either BspD-3×FLAG, which localizes to the ER when expressed ectopically in HeLa cells without affecting host secretion, or BspE-3×FLAG, a VirB-dependent effector that does not target the secretory pathway (Fig. 7D). Taken together and consistent with our ectopic expression data (Fig. 5), these results indicate that bacterially-expressed BspA also affects the host secretory pathway in infected cells.

### *Brucella* inhibition of host secretion precedes rBCV biogenesis and replication

While the above results demonstrate that *Brucella* inhibits host secretion at 30 h pi, *i.e.* when the bacteria replicate within ER-derived rBCVs, we next sought to examine whether they also exert an inhibitory effect on host secretion prior to reaching the ER, *i.e.* when VirB-associated functions control BCV trafficking [8], [14], [16]. When SEAP secretion was measured at 8 h pi, *i.e.* before bacteria generate rBCVs and replicate, infection with the wild-type strain 2308 inhibited SEAP secretion to levels comparable to those seen at 30 h pi (Fig. 7C). Deletion of either *bspB* or *bspF*, but not *bspA*, relieved secretion inhibition, which was restored upon genetic complementation of the Δ*bspB* or Δ*bspF* mutants (Fig. 7C), as observed at 30 h pi (Fig. 7A–B). Additionally, infection with a Δ*virB9* mutant, which was interpretable at this time point due to its similar intracellular viable numbers to 2308 (Fig. S3), also failed to inhibit SEAP secretion (Fig. 7C), consistent with the roles of BspB and BspF in this process. Altogether, these results demonstrate that *Brucella*-mediated inhibition of host secretion is a VirB-dependent process that requires BspB and BspF and occurs prior to rBCV biogenesis.

### Secretion-interfering Bsp effectors contribute to *Brucella* intracellular growth and persistence

Given the effect of BspB, BspF and BspA on the host secretory pathway and the importance of this compartment for *Brucella* intracellular trafficking and replication, we sought to determine whether these proteins play a role in *Brucella* pathogenesis. First, intracellular growth of the single and triple deletion mutants was examined in murine BMMs by CFU enumeration over 24 h.

**Figure 8:**
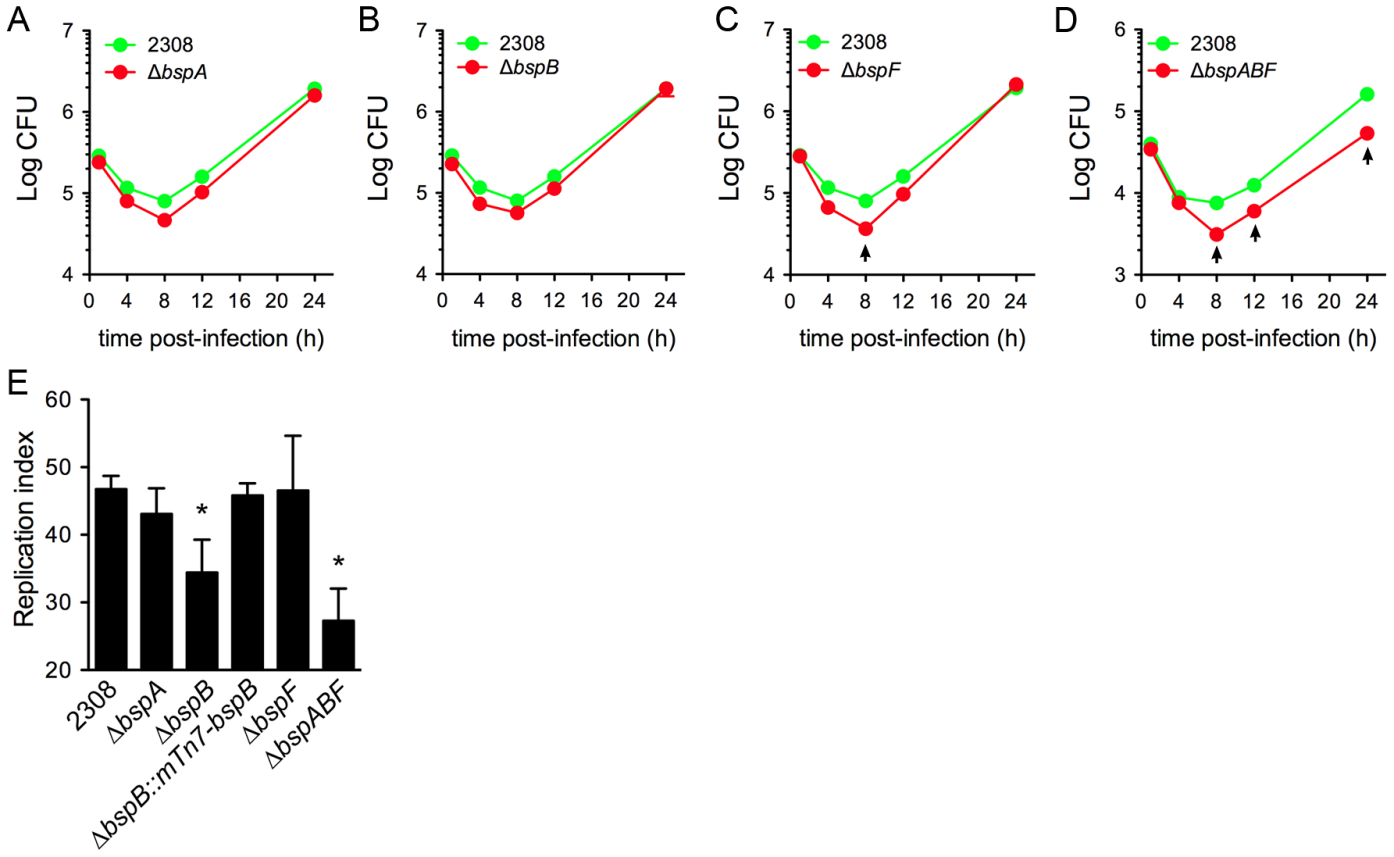
Contribution of BspA, BspB and BspF to *Brucella* intracellular growth. (A–D) Intracellular growth of *B. abortus* 2308 and its isogenic in-frame deletion mutants Δ*bspA* (A), Δ*bspB* (B), Δ*bspF* (C) and Δ*bspABF* (D) in BMMs. Cells were infected and intracellular CFUs were enumerated at various time points. Data shown are means ± SEM from a representative experiment performed in triplicates. (E) Single-cell analysis of *Brucella* replication in BMMs infected with either *B. abortus* 2308, its isogenic mutants Δ*bspA*, Δ*bspB*, Δ*bspF* and Δ*bspABF* or the complemented strain Δ*bspB*::*mTn7-bspB*. Data is represented as the percentage of infected cells supporting bacterial replication and are means ± SD of three independent experiments.

No significant difference was observed in *Brucella* replication between the wild type strain and the Δ*bspA* or Δ*bspB* mutants, as similar numbers of CFUs were recovered during the time course of infection, while the Δ*bspF* mutant showed decreased survival by 8 h pi but underwent subsequent replication similar to the wild type strain (Fig. 8A–C). In contrast, a stronger decrease in recoverable bacteria was observed at 8 h pi and afterwards for the triple Δ*bspABF* mutant compared to the wild type strain (Fig. 8D), indicating that the combined deletion of several Bsp effectors affects *Brucella* intracellular growth. Since CFU enumeration examines global intracellular growth within a population, it may lack the ability to uncover minor, yet significant, phenotypic defects at the cellular level. To evaluate the intracellular growth of the *bsp* mutants by single cell analysis, we examined bacterial replication in BMMs at 24 h pi using immunofluorescence microscopy. Under infection conditions where 1–4 bacteria were initially phagocytozed by BMMs (data not shown), the percentage of infected cells supporting bacterial replication was scored as those containing at least 10 bacteria and showed that the Δ*bspB* mutant strain displayed a significant replication defect when compared to the wild type strain, which was genetically complemented (Fig. 8E). Consistent with the CFU enumeration (Fig. 8D), the Δ*bspABF* mutant was also significantly impaired in replication inside BMMs (Fig. 8E), confirming its intracellular growth defect. Hence, both BspB and the coordinated action of BspA, BspB and BspF promote *Brucella* intracellular replication.

To extend these findings and examine whether the intracellular defects of these mutants translate into virulence defects, we monitored establishment and persistence of these strains in a BALB/c mouse model of chronic brucellosis. Following intraperitoneal inoculation with either the wild type 2308 strain, the Δ*bspA*, Δ*bspB*, Δ*bspF* or Δ*bspABF* deletion mutants, splenomegaly was assessed as a readout for inflammation, and bacterial loads enumerated in the spleen and liver at day 3, 7 and 42 pi, to assess initial infection (day 3), establishment of the chronic stage (day 7) and persistence (day 42). In this infection model, a Δ*virB9* mutant did not induce any splenomegaly and failed to establish a chronic infection and persist in both spleen and liver (Fig. S9). Although all strains induced splenomegaly similarly and colonized the spleen equally at all times points, the numbers of recoverable Δ*bspB* and Δ*bspABF* mutants from the liver at day 42 pi were significantly lower than the wild type strain (Fig. S10), indicating a role of BspB and the cumulative functions of BspA, BspB and BspF in bacterial persistence in this organ.

## Discussion

Many Gram-negative bacterial pathogens including *Brucella* spp., *Legionella pneumophila*, *Coxiella burnetii*, *Bartonella* spp., *Helicobacter pylori*, *Bordetella pertussis*, and *Rickettsia prowazekii* use T4SS to translocate effector proteins into host cells as part of their pathogenic process [42]. The identification and characterization of these proteins is a crucial step in our molecular understanding of many human diseases and has been the subject of several bioinformatics-based efforts with recent successful outcomes in the case of *Legionella*, *Coxiella*, *Bartonella* and *Brucella* [19], [20], [22], [23], [26], [43]. Here we have used similar approaches and protein translocation reporter systems to identify in *Brucella abortus* 11 translocated proteins (BspA to K), 10 of which are novel since BspI (BPE865) has previously been reported [20], and at least 5 are translocated in a VirB T4SS-dependent manner. These findings expand the number of known *Brucella* effector proteins to 19, including VceA and VceC [19], RicA [24], and 6 BPEs [20] and suggest that this pathogen translocates into host cells an array of proteins with possibly redundant functions.

The initial demonstration of protein translocation of the Bsp effectors used a TEM1 β-lactamase reporter system, in which we systematically tested both N- and C-terminal TEM1 fusions. This was a necessary precaution due to a lack of consensus for *Brucella* VirB-mediated translocation signals, as exemplified by the findings that the N-terminal region of BPE123 is necessary for its translocation into host cells [20] while the C-terminal region of VceC is required for efficient secretion [19]. Interestingly, only the C-terminal TEM1 fusion proteins to BspB, BspC, BspD and BspE and only the N-terminal TEM1 fusions to BspG and BspK showed translocation, while those to BspA, BspF, BspH, BspI and BspJ showed translocation regardless of the TEM1 tag location. This suggests that the position of the reporter tag may interfere with translocation signals and protein translocation. Consequently, one cannot exclude that TEM1 tagging at either end of the negative candidate effector proteins also interfered with their translocation or led to fusion proteins in which TEM1 activity was impaired. These observations highlight the necessity to characterize the molecular mechanisms by which the *Brucella* VirB T4SS translocates proteins into host cells and the caveats associated with using reporter translocation systems.

A retrospective analysis of the criteria used to identify the Bsp proteins did not reveal consensus features shared by these effectors, including some that would typify VirB substrates. While BspA, BspB, BspC, BspD, BspH and BspK all possessed a RXR (or RXK in the case of BspC) motif in their C-terminus (Table S2), reminiscent of the *A. tumefaciens* Arginine-rich motifs in VirB effectors [25], this could not be strictly correlated with VirB-dependency or a requirement for a free C-terminus for translocation in our assays, nor was the net charge of their C-termini. Except for BspC, none of the Bsp proteins contained a predicted Sec-dependent signal peptide sequence, suggesting that their translocation does not require Sec-dependent translocation into the periplasm. Most *bsp* genes nonetheless showed a significant deviation in their GC content from that of the total genome (Table S2). Despite the intracellular lifestyle of *Brucella* spp., which intuitively limits genetic exchange with other bacteria, and their overall genetic homogeneity, this suggests that these *bsp* genes have been acquired via horizontal transfer during evolution of an ancestral genome, perhaps in extracellular environments, in agreement with evidence for discrete horizontal transfer events gained from genomic comparisons between *Brucella* spp. [44]. Consistently, the colinear genetic organization of *bspH* and *bspI* adjacent to an IS2020 transposase-encoding open reading frame (BAB1_1863) argues for the presence of a mini genomic island carrying these effectors.

A CyaA translocation reporter assay with sufficient sensitivity to detect *Brucella* effector translocation at 6 h pi identified BspA, BspB, BspC, BspE and BspF as VirB T4SS substrates, since their translocation was significantly reduced in the Δ*virB9* mutant background. In contrast, the translocation of BspG and BspK was independent of a functional T4SS, suggesting the presence of another unknown translocation system in *Brucella*. This is in line with the VirB-independent translocation of BPE865 (BspI) and BPE159 [20]. Of note, translocation of the BspI-CyaA (BPE865) was VirB-dependent under our experimental conditions, unlike a previous report by Marchesini *et al.* [20]. Although the same CyaA-based translocation assays were used in both studies, we found that normalization of cAMP levels to recoverable CFUs was essential to uncover VirB dependency in these assays, a technical variation that could explain the contrasting results obtained. Surprisingly, the translocation of the BspH, BspI and BspJ was VirB-dependent when tagged at the C-terminus, indicating these proteins are VirB T4SS substrates, but VirB-independent when tagged on their N-terminus. These results suggest that these proteins may be promiscuously translocated by more than one translocation system, the VirB T4SS and another yet unknown secretion system, through translocation signals that confer pathway specificity and are differentially affected by the CyaA tag location. Translocation of bacterial effectors via multiple type three secretion systems (T3SS) and the flagella export system has been shown for YplA in *Yersinia* [45] *and* SptP in *Salmonella* [5]. *Brucella* does possess all the flagellar genes needed for the assembly of a functional flagellum, but not those encoding for chemotaxis [46], [47]. Considering that flagellar and *virB* genes are co-regulated in *Brucella* [48], some of these *Brucella* secreted proteins could also be targeted to and translocated by the flagella export pathway, a hypothesis that remains to be tested. Additionally, the secretion of RicA in broth culture in a VirB T4SS- and flagella-independent manner supports the possibility of yet another unidentified translocation system in *Brucella* [20], [24].

With the exception of VceC, shown to induce ER stress and secretion of proinflammatory cytokines [21], the recently discovered *Brucella* effectors remain uncharacterized and virulence phenotypes associated with individual effector proteins have not been observed, presumably due to some functional redundancy [20]. Additionally, the role of *Brucella* effectors in the biogenesis and trafficking of the BCV along the endocytic and secretory pathway remains poorly understood. RicA was shown to interact with Rab2, a small GTPase recruited on BCVs and required for *Brucella* intracellular replication [15], potentially assigning a direct role for RicA in the intracellular trafficking of *Brucella* [24]. Yet, how RicA interaction with Rab2 contributes to the intracellular lifestyle of *Brucella* remains speculative. In our attempt to characterize the functions of the newly identified *Brucella* effectors, a high proportion of the Bsp proteins localized to compartments of the secretory pathway when ectopically expressed in HeLa cells. BspA, BspB, BspD and BspK localized to the ER and BspC localized to the Golgi apparatus. Considering the importance of the secretory pathway in *Brucella* intracellular pathogenesis, where BCVs engage early secretory compartments by initiating interactions with ERES [14] and recruit Rab2 and GAPDH [15], we pursued these initial observations to demonstrate that BspA, BspB and BspF specifically inhibit host protein secretion and membrane trafficking along the secretory pathway. That BspF affects host protein secretion despite a cytosolic and plasma membrane localization is not inconsistent, as i) the bacterially-translocated form may localize differently via interactions with other bacterial effectors or BCV-associated host factors, and ii) various host cell proteins that mediate membrane trafficking events localize to the cytosol and are targeted to vesicular compartments via regulatory proteins [49]. A number of T4SS and T3SS bacterial effectors in other pathogens also affect functions of the secretory pathway. When overexpressed in eukaryotic cells, the *L. pneumophila* Dot/Icm effectors DrrA/SidM [50], [51], and LidA [52] disrupt the Golgi apparatus, and RalF [53], AnkX [23], the *Coxiella* Dot/Icm effector CBU0635 [54], and the Enteropathogenic *Escherichia coli* (EPEC) T3SS effector NleA [55] inhibit host protein secretion, similar to the effect of BspA, BspB or BspF. Unlike the *L. pneumophila* Dot/Icm effector proteins that cause Golgi apparatus fragmentation [50], [52], the overexpression of BspA, BspB or BspF did not alter the morphology of secretory compartments including the ERES, ERGIC, and Golgi apparatus, suggesting subtle effects on this compartment. We therefore speculate that the effect these effectors exert on protein secretion results from discrete interference with specific molecular processes along the secretory pathway and may reflect a specific inhibitory role in protein secretion related to *Brucella* pathogenesis, and/or a collateral consequence of BCV trafficking-related modulation of membrane transport. Importantly, inhibition of host protein secretion and secretory membrane trafficking was also revealed during *Brucella* infection and required the functions of BspB and BspF, functionally correlating effector ectopic expression and infection, as previously reported only in the case of EPEC NleA [55]. Contrary to the effect of ectopically-expressed BspA and unlike BspB and BspF, deletion of *bspA* did not restore secretion in *Brucella*-infected cells, suggesting some functional redundancy between BspA and other translocated *Brucella* proteins, otherwise absent in the ectopic expression model used. Yet, BspA overexpression in *Brucella* enhanced inhibition of host secretion, arguing for a role of this effector in also impairing secretory functions in the context of an infection, as seen with ectopically-expressed BspA.

While our results clearly assign modulatory functions along the secretory pathway to the VirB T4SS effectors BspA, BspB and BspF, we also provide evidence that they contribute to biogenesis of rBCVs and bacterial replication in macrophages, and long-term persistence in the liver of chronically infected mice. Given that combining deletions in *bspA*, *bspB* and *bspF* resulted in additive effects on restoration of inhibition of secretion by *Brucella* and intracellular replication defects, we also speculate that these effectors coordinately act on modulating the secretory pathway to promote *Brucella* pathogenesis. Collectively, our results potentially link VirB T4SS effector-mediated modulation of the secretory pathway with BCV trafficking, although we cannot yet conclude whether BspA-, BspB- and BspF-mediated inhibition of host protein secretion is a requirement for rBCV biogenesis and bacterial replication, or is unrelated to their requirement for optimal intracellular growth. Additionally, *Brucella*-mediated inhibition of host protein secretion may affect secretion or surface exposure of immunity-related molecules to the pathogen's benefit. Consistent with our results, *Brucella* induces retention of MHC Class I molecules in the Golgi apparatus in human macrophages, resulting in downmodulation of CD8^+^ cytotoxic T-cell responses [56], possibly via BspA, BspB and/or BspF. Similarly, it will be most interesting to examine whether these effectors downmodulate secretion of pro-inflammatory cytokines, an effect that may explain the persistence defects of the Δ*bspB* and Δ*bspABF* mutants observed in the liver.

By identifying novel VirB T4SS effectors and characterizing their activities and contribution to pathogenesis, this study expands our knowledge at the molecular level of how *Brucella* manipulates the host secretory pathway to promote its intracellular survival. Future studies focusing on the mechanisms of BspA, BspB and BspF actions and on the characterization of the additional Bsp proteins identified should reveal new facets of *Brucella* molecular interactions with host cells, generating much-needed knowledge about its pathogenic mechanisms.

## Materials and Methods

### Reagents

Antibodies were from the following sources: mouse anti-Beta-lactamase (QED Bioscience, Inc.), mouse monoclonal anti-Omp19 SC10 and anti-p17 (gifts from Dr Axel Cloeckaert, INRA, France), mouse anti-CyaA 3D1(Santa Cruz Biotechnology, Inc.), mouse monoclonal anti-HA, mouse monoclonal anti-FLAG M2 and mouse anti-SEAP (Sigma-Aldrich Co. LLC.), rabbit polyclonal anti-GFP (Life Technologies), rabbit anti-Calnexin (Enzo Life Sciences), mouse anti-GM130 (BD Transduction Laboratories), rabbit anti-COPII (Thermo Scientific, Pierce Antibodies), mouse anti-ERGIC-53 (Enzo Life Sciences), goat anti-*Brucella* LPS (gift from Dr Renée Tsolis, UC Davis, CA), rat anti-mouse LAMP-1 (clone 1D4B, developed by J. T. August and obtained from the Developmental Studies Hybridoma Bank (DSHB) developed under the auspices of the NICHD and maintained by the University of Iowa, Department of Biological Sciences, Iowa City, IA 52242), rabbit polyclonal anti-Actin (Bethyl Laboratories, Inc.), rabbit anti-BiP and mouse monoclonal anti-CHOP (Cell Signaling Technology); Alexa Fluor 488-donkey anti-mouse, anti-rat antibodies (Invitrogen, Life Technologies), or Cyanin 5-conjugated goat anti-rabbit and anti-mouse (Jackson ImmunoResearch Laboratories, Inc.). HRP-conjugated anti-rabbit IgG or anti-mouse IgG (1∶10,000, Cell Signaling Technology) and the ECL western blotting substrate (Thermo Scientific, Pierce Protein Biology Products) were used for Western blotting. DAPI (Invitrogen, Life Technologies) was used for DNA staining. Dulbecco's modified Eagle medium (DMEM), DMEM without phenol red, phosphate saline buffer (PBS), Hank's Balanced Salt Solution (HBSS), fetal bovine serum (FBS) and gentamicin were from Life Technologies. FuGENE HD and X-tremeGENE 9 transfection reagents were used to transfect cell lines according to the manufacturer's instructions. SEAP secretion assays were performed with the SEAP reporter gene assay, chemiluminescent kit (Roche Applied Science). The levels of intracellular cAMP were measured with the cAMP Enzyme Immunoassay kit from Sigma-Aldrich Corporation LLC while the beta-lactamase activity was assayed with the Beta-lactamase loading solution and Probenecid from Life Technologies (Invitrogen). ER stress measurements were implemented with the Cignal ERSE Reporter Luciferase assay kit from SABiosciences. Luciferase measurements were performed with the Passive Lysis Buffer and Dual-Luciferase Reporter Assay System from Promega Corporation. Tunicamycin, Cycloheximide and Brefeldin A were purchased from Sigma-Aldrich.

### Bacterial strains and culture

*Brucella abortus* 2308 strains and derivatives were grown on tryptic soy agar (TSA) (Difco) for 72 h at 37°C or in tryptic soy broth (TSB) at 37°C with shaking overnight to late logarithmic phase. *B. abortus* strains 2308Δ*virB9* [14] and DsRed_m_-expressing 2308 (pJC44) [13] have been described previously. *Brucella* strains harboring pJC120, pJC121, pJC125 and pJC126 plasmids described in Table S2 were cultured in TSA or TSB supplemented with kanamycin (50 µg/ml). All manipulations of *B. abortus* strains were performed in a Biosafety Level 3 facility according to standard operating procedures approved by the Rocky Mountain Laboratories Institutional Biosafety Committee and in compliance with the CDC Division of Select Agents and Toxins regulations. *Escherichia coli* strains were grown in Luria-Bertani (LB) broth at 37°C, supplemented with 50 µg/ml of kanamycin and 100 µg/ml of ampicillin when necessary.

### Plasmids

Primers and plasmids used in this study are described in Table S3. The pSEAP-2 control vector, pCMV-HA, pEGFP-C1 and pEGFP-N1 were purchased from Clontech. Plasmids pHA-ARF1[T31N] and pGFP-ARF1[T31N] have been described [30] and pEGFP-VSVGts045 was a gift from Dr Jennifer Lippincott-Schwartz (NIH, Bethesda, MD). The β-lactamase TEM-1 (pJC120 and pJC121) and CyaA (pJC125 and pJC126) fusion plasmids were constructed from pBBR1MCS-2 [57]. First, the *lac* promoter region of pBBR1MCS-2 was replaced with a *lacI^q^-Ptrc* fragment by ligating *Xho*I-digested pBBR1MCS-2 to a PCR product from plasmid pTrc99A [58] obtained with primers TW331 (CGGGCCCCCCCTCGAGCCGCCAACACCCGCTGAC) and TW332 (TACCGTCGACCTCGAGCATTATTACCACCTCCTCTG) using the In-Fusion PCR Cloning System (Clontech). The modification was confirmed by sequencing. Vectors pJC120 and pJC121 were then constructed by cloning PCR products containing the *blaM* gene from pCX340 [27] with primers TW333 (GCTTGATATCGAATTCCACCCAGAAACGCTGGTGAAAG) or TW340 (CGGGCTGCAGGAATTCCCAATGCTTAATCAGTGAGGC), and TW333 and TW334 (CGGGCTGCAGGAATTCTCACCAATGCTTAATCAGTGAGGC) respectively, into the *Eco*RV-digested pBBR1-*lacI^q^*-*Ptrc* derivative using In-Fusion, and were confirmed by sequencing. The resulting plasmids pJC120 and pJC121 encode the mature form of TEM-1 under the control of the *lacI^q^* gene and the IPTG-inducible P*trc* promoter, allowing for cloning of potential effector protein genes with *blaM* to generate effector-TEM-1 fusion proteins. The stop codon of the *blaM* gene was removed in pJC120 to allow generation of in-frame N-terminal fusions, while it was left intact in pJC121 to allow generation of in-frame C-terminal fusion proteins. The plasmids pJC123 and pJC124 were constructed by PCR amplifying the 3×FLAG sequence from a synthesized template with primers TW337 (GCTTGATATCGAATTCGATTATAAAGATGATGATG), TW338 (CGGGCTGCAGGAATTCCTATTTATCATCATCATCT), and TW337, TW343 (CGGGCTGCAGGAATTCTTTATCATCATCATCT), respectively, into the *Eco*RI-digested pBBR1-*lacI^q^*-*Ptrc* plasmid using In-Fusion. Inserts were confirmed by sequencing.

The plasmids pJC125 and pJC126 were constructed by PCR amplification of the catalytic portion of the *cyaA* gene (first 399 codons) from plasmid pJB2581 [59] with primers TW682 (CGGTATCGATAAGCTTCAGCAATCGCATCAGGCTGGT) or TW684 (ATTCGATATCAAGCTTCTGGCGTTCCACTGCGCCCAGCGA), and TW682 and TW683 (ATTCGATATCAAGCTTTCACTGGCGTTCCACTGCGCCCAGCGA), respectively, and cloning into the *Hin*dIII-digested pBBR1 *lacI^q^*-*Ptrc* derivative using In-Fusion, and were confirmed by sequencing. The stop codon in the *cyaA* open reading frame in plasmid pJC125 was removed to allow for generation of N-terminal in-frame fusions of potential effector-CyaA fusions, while it was left intact in pJC126 to allow for generation of in-frame C-terminal fusions. The resulting plasmids: pJC120, pJC121, pJC123, pJC124, pJC125 and pJC126 were used to construct either TEM-1, 3×FLAG or CyaA *Brucella* effector fusions (Table S3). Full-length genes encoding putative *Brucella* effectors to be fused to either the *blaM* or *cyaA* fragments were amplified by PCR using genomic DNA from *B. abortus* 2308 strain as a template with sequence specific primers containing restriction sites for cloning into either pJC120 (*Xho*I), pJC121 (*Sal*I), pJC125 (*Eco*RI) or pJC126 (*Sal*I)) vectors (Table S3) using the In-Fusion PCR Cloning System (Clontech, 639650).

To tag putative *Brucella* effectors with either GFP or HA, open reading frames of the *Brucella* target genes were amplified by PCR using genomic DNA from *B. abortus* 2308 strain as template, Platinum *Pfx* DNA Polymerase (Invitrogen), and specific primers (Table S3). The PCR-amplified DNA fragments were cloned into either *Bgl*II-digested pEGFP-C1 (Clontech), *Pst*I-digested pEGFP-N1 (Clontech), or *Eco*RI-digested pCMV-HA (Clontech) using the In-Fusion PCR Cloning System (Clontech). The identity and orientation of all constructs generated by PCR were confirmed by restriction digest analysis, sequencing and protein expression where indicated.

### *Brucella* in-frame deletion mutants and genetic complementation

In-frame deletion mutants of *B abortus* strain 2308 were generated using the SacB-assisted allelic replacement suicide vector pJC80, as described previously [14], and were designed to preserve the integrity of the downstream genes and avoid any polar effect of the deletion. Recombinant in-frame deletions of either BAB1_0678 (*bspA*), BAB1_0712 (*bspB*), BAB1_0847 (*bspC*), BAB1_1611 (*bspD*), BAB1_1671 (*bspE*), BAB1_1948 (*bspF*), BAB_10227 (*bspG*) and BAB2_0541 (*bspK*) were constructed by fusing 5′ and 3′ fragments flanking the open reading frame of the gene using PCR amplification and the relevant primers (Table S4). The 5′ fragments contained the upstream region, the start codon and the first 1 to 3 codons of each gene. The 3′ fragments contained the downstream region, the last 1 to 3 codons of each gene, and the stop codon. 5′ and 3′ fragments were fused and cloned into the *Bam*HI and *Sal*I sites of pJC80 using the In-Fusion PCR Cloning System (Clontech) and fully sequenced. To perform allelic replacement in the chromosome of *B abortus* 2308 strain, deletion plasmids were introduced by electroporation, carbenicillin-resistant, sucrose-sensitive electroporants were selected and sub-cultured further in TSB. Cultures were subjected to sucrose counter-selection by plating onto TSA plates supplemented with 5% sucrose (wt/vol) in order to isolate clones that had undergone allelic replacement. The presence of the deleted allele within the correct chromosomal locus was verified by PCR using primers listed in Table S3. Independent clones carrying the correct in-frame deletion were isolated and used for further studies. The multiple deletion mutant Δ*bspABF* was generated by sequential allelic replacement.

The *B. abortus* 2308 Δ*bsp*B and Δ*bsp*F in-frame deletion mutants were complemented using a derivative of the mini-Tn*7* system. First, the plasmid pmTn*7*K was constructed by amplifying a 937 bp fragment from pBBR1MCS-2 containing the *aphA3* gene and its own promoter region using primers RC7 and RC8 (Table S3) and cloning into pUC18Tmini-Tn*7* [60] digested with *Eco*RV using the In-Fusion PCR Cloning System, in order to generate a mini-Tn*7* derivative expressing kanamycin resistance. The *bspB* and *bspF* genes and their respective promoter regions were subsequently cloned into pmTn*7*K using *Spe*I and *Bam*HI. Briefly, the 561 bp *bspB* gene was amplified using primers RC389 and RC390 (Table S3), and a 150 bp fragment upstream of BAB1_0710 was amplified using primers RC360 and RC388 (Table S2). The two fragments were fused by overlap PCR and cloned into pmTn*7*K to generate pmTn*7*K-*bspB* using the In-Fusion PCR Cloning System. A fragment containing the 1283 bp *bspF* gene and 340 bp upstream of its start codon were PCR amplified using primers RC395 and RC396 (Table S3) and inserted into pmTn*7*K to create pmTn*7*K-*bspF*. All inserts were confirmed by sequencing. The resulting pmTn*7*K-*bspB* and pmTn*7*K-*bspF* plasmids were introduced into the Δ*bspB* and Δ*bspF* mutants, respectively, together with the helper plasmid pTNS2 [60] by electroporation. Electroporants were selected on TSA plates containing 50 µg/ml of kanamycin, and clones carrying the mTn*7*K derivatives inserted within the *att*Tn*7* site downstream of the BAB2_0658 *glmS* gene were confirmed using PCR primers RC603 (ATCATCCTCATCACCGACAA) and RC604 (GCTATATTCTGGCGAGCGAT).

### Mammalian cell culture, transfections and infections

Murine bone marrow-derived macrophages (BMMs) from 6–12 week-old female C57BL/6J mice (Jackson Laboratories, Bar Harbor, ME) were generated and cultured as described before [61]. HeLa cells (ATCC clone CCL-2) were cultured as described [13]. J774.A1 macrophage-like cells (ATCC, TIB-67) were maintained in Dulbecco's high glucose DMEM supplemented with 10% FBS and 4 mM L-glutamine at 37°C in 7% CO_2_. Human Embryonic Kidney 293T (HEK293T; ATCC CRL-11268) were cultured in Dulbecco's Modified Eagle's Medium (DMEM) supplemented with 10% FBS at 37°C in 7% CO_2_. HeLa and HEK293T cells were transfected with either FuGENE HD or X-tremeGENE 9 transfection reagents (Roche Applied Science) according to the manufacturer's instructions at a ratio of 3∶1 (reagent∶DNA; v/w).

HeLa cells and BMMs were infected with *Brucella* strains at the appropriate multiplicity of infection (MOI) as previously described [13]. For experiments that required incubation at 40°C or 32°C, pre-warmed media was employed at each step. Temperature shifts from 40°C directly to 32°C were performed by removing the 40°C medium and replacing it with medium at 32°C. For infections of J774.A1 cells, two days before infection, cells were seeded at either 8×10^3^ (96-well plates) or 5×10^4^ (24-well plates) cells/well in complete medium, then infected with *Brucella* strains in triplicate wells at the desired MOI. Following 30 min of incubation at 37°C, cells were washed five times with pre-warmed DMEM and incubated for another 30 min before a 1 h treatment with 100 µg/ml gentamicin to kill residual extracellular *Brucella*. Intracellular bacterial growth was evaluated in triplicates by lysing infected cells with 0.1% Triton X-100 in H_2_O at the indicated times after infection and plating a series of 1∶10 dilutions on TSA plates for colony-forming unit (CFU) enumeration.

### TEM1 protein translocation assay

The translocation of translational fusions between TEM1 and the *Brucella* candidate proteins described in Table S1 was evaluated by detecting β-lactamase activity in infected J774.A1 cells as previously described [19]. TEM1 fusions described in Table S3 were transformed in *Brucella* by electroporation and the expression of the fusions was verified by Western blot analysis with an anti-β-lactamase antibody (1∶1000, QED Bioscience Inc, San Diego, CA). Bacteria were grown in TSB supplemented with 50 µg/ml of kanamycin overnight, then treated with 1 mM IPTG for 2 h to induce expression of fusion proteins before infection. J774.A1 cells were then infected with *Brucella* strains harboring the TEM1 fusions at an MOI of 1000 in presence of 0.1 mM of IPTG throughout the infection. This MOI was necessary to achieve high levels of infection within the population (>95% infected cells, data not shown) and within individual cells and detect TEM fusion translocation. Infected cells were centrifuged at 400× *g* for 10 min to initiate bacterial-cell contact followed by incubation at 37°C for 30 min after which the cells were washed 5 times and incubated for another 30 min. At 1 h pi, cells were treated with gentamicin for 1 h to kill extracellular bacteria. At 16 h pi, cells were washed two times in DMEM and loaded with the fluorescent substrate CCF2/AM (LiveBLAzer-FRET B/G loading kit; Invitrogen) in the β-lactamase loading solution supplemented with 15 mM Probenecid (Invitrogen). Cells were incubated in the dark for 90 min at room temperature and then observed under epifluorescence using a Carl Zeiss Axiovert 200 M fitted with a β-lactamase Blue/Aqua 41031 filterset (Chroma Technology Corp.). At least 300 cells were counted in triplicate wells to determine the percentage of cells emitting a blue fluorescence (TEM1-positive). The presented data are mean values ± SD from three independent experiments performed in triplicate. In each experiment, cells were then fixed and stained for *Brucella* (see below) to verify that >95% cells were infected, ensuring that the CCF2/AM conversion events occurred in infected cells.

### CyaA protein translocation assay

CyaA adenylate cyclase protein translocation assays were performed as previously described [20], [28] with the following modifications. CyaA fusion constructs described in Table S3 were introduced into either the wild type *B. abortus* 2308 strain or its isogenic Δ*virB9* mutant [14] by electroporation, and their expression was detected by Western blot analysis using monoclonal anti-CyaA 3D1 antibody (Santa Cruz Biotechnology, Inc.). Bacteria were grown in TSB supplemented with 50 µg/ml of kanamycin overnight and treated with 1 mM IPTG for 2 h before infection. J774.A1 cells were infected at an MOI of either 1000 (wild type strains) or 5000 (Δ*virB9* strains). These MOIs were necessary to achieve high levels of infection that allowed for detection of cAMP production. Infected cells were centrifuged at 400× *g* for 10 min followed by incubation at 37°C for 30 min after which the cells were washed 5 times and incubated for another 30 min. At 1 h pi, cells were treated with gentamicin for 1 h to kill extracellular bacteria. At 6 h post infection, cells were lysed and processed for cAMP levels using a colorimetric direct cAMP Enzyme Immunoassay Kit (Sigma, CA200) according to the manufacturer's instructions. Each independent experiment was performed in triplicates and the levels of cAMP were normalized to the number of intracellular bacteria (CFUs). Data are means ± SD from three independent experiments.

### Immunofluorescence microscopy

For immunofluorescence staining, cells cultured on 12 mm glass coverslips in 24-well plates or in 96 well plates were fixed in 3% paraformaldehyde in PBS, pH 7.4 for 20 min at 37°C and processed for immunostaining as described previously [13]. Samples were observed with a Carl Zeiss MicroImaging AxioImager epifluorescence microscope for quantitative analysis or a LSM710 confocal laser scanning microscope for image acquisition (Carl Zeiss Micro Imaging, Thornwood, NY). Representative confocal micrographs of 1024×1024 pixels were acquired and assembled for presentation using Adobe Photoshop CS3.

### SEAP secretion assay

SEAP secretion assays were performed as previously reported [62] with some modifications. Briefly, HEK293T cells were seeded in 24-well plates to ∼60% confluency and co-transfected with plasmids expressing the indicated *Brucella* secreted proteins (300 ng DNA) and the secreted embryonic alkaline phosphatase (SEAP) (200 ng DNA). At 16 h post transfection, cells were washed and fresh tissue culture medium was added. Eight hours later, media containing culture supernatant (extracellular SEAP) was removed to collect secreted SEAP and the cell associated (intracellular) SEAP was harvested by washing cells once with PBS and lysing them with equal volume of culture medium containing 0.5% Triton X-100. SEAP activities were measured in triplicate wells using SEAP reporter gene assay, chemiluminescent kit (Roche Applied Science). Data are presented as the SEAP secretion index, which is a ratio of extracelluar SEAP activity to intracellular SEAP activity normalized to values obtained from cells co-transfected with empty vector controls. Each experiment was carried out in triplicate and at least three independent experiments were performed.

For SEAP secretion measurements in infected cells, HeLa cells were plated in 24-well plates at 5×10^4^ cells/well. Sixteen hours after transfection, cells were infected with the indicated *Brucella* strains as previously described [13]. At 24 h post infection, all wells were treated with 5 µg/ml Brefeldin A (BFA) and 10 µg/ml of cycloheximide for 30 min to reversibly block secretion and novel SEAP biosynthesis and synchronize SEAP secretion. BFA was then removed with 5 washes using DMEM and subsequent incubation in complete medium containing 10 µg/ml cycloheximide to allow reconstitution of the secretory pathway and secretion of the SEAP pool. SEAP activity was measured in quadruplicate at indicated times post BFA washout and independent experiments were performed at least three times. Staining of the Golgi apparatus of cells grown on glass coverslips using a mouse anti-GM130 antibody was performed to evaluate the kinetics of secretory pathway reconstitution after BFA washout in uninfected and *Brucella*-infected cells.

### VSV-G trafficking assay

HeLa cells were co-transfected with equal amounts (0.5 µg) of VSV-Gts045-GFP [63], [64] and plasmids expressing HA-tagged *Brucella* effectors and incubated at 37°C for 4 h to allow transfection to occur. Cells were then moved to 40°C and incubated for 16–20 h at this non-permissive temperature to allow for VSV-Gts045-GFP accumulation in the ER. Cells were replenished with fresh medium containing 25 mM HEPES pH 7.4 and 10 µg/ml cycloheximide (Sigma) to stop further protein synthesis, and incubated for an additional hour at 40°C. Transfected cells were then moved to 32°C (permissive temperature) to allow VSV-Gts045-GFP to fold properly and traffic along the secretory pathway and then fixed and analyzed by immunofluorescence microscopy. In the case of *Brucella* infections, HeLa cells were first infected with *Brucella* for 24 h at 37°C, then transfected to express VSV-Gts045-GFP for 20 h at 40°C and further processed as above. Quantitative data of VSV-Gts045-GFP transport are means ± SD from three independent experiments.

### Measurement of ER stress

To evaluate ER stress, the transcriptional activity of the ER stress element (ERSE) was measured using the Cignal ERSE Reporter Luciferase Assay Kit (SA Biosciences, Qiagen, Frederick, MD). The ERSE reporter assay is a mixture of an ERSE-responsive luciferase construct and a constitutively expressed *Renilla* luciferase construct (40∶1). Following the manufacturer's instructions, HEK293T cells expressing HA- or GFP-tagged *Brucella* effectors were transfected in 96-well plates with either a ERSE reporter plasmid, a negative control, or a positive control (a mixture of constitutively expressing GFP, constitutively expressing firefly luciferase, and constitutively expressing Renilla luciferase constructs (40∶1∶1)) for 24 h according to the manufacturer's instructions. The ER stress inducer Tunicamycin (5 µg/ml) was used as a positive control. At 32 h post-transfection, cells were washed with Dulbecco's PBS and harvested in 100 µl of Passive Lysis Buffer (Promega) and used to measure luciferase activity. The luciferase assay was developed with the Dual-Luciferase Reporter Assay System (Promega) according to the manufacturer's instructions. Bioluminescence was detected using a Tecan Infinite M1000 luminometer (Tecan Group LTD). Data was processed by dividing the luminescence intensity of firefly luciferase by that of *Renilla* luciferase, to calculate the “relative luciferase activity” as the luminescence ratio of the test plasmid to that of the reporter-control. Experiments were performed in triplicates and repeated at least three times and the standard deviation is indicated. Expression levels of BiP and CHOP were determined by Western blot analysis using either an anti-BiP monoclonal antibody or a mouse anti-CHOP monoclonal antibody (1∶1,000). The membranes were stripped and reprobed using an anti-Actin antibody (1∶25,000) to verify equal loading.

### Western blot analysis

Bacterial or mammalian cell lysates were generated using cell lysis buffer 10× (Cell Signaling Technology, Inc). Samples were normalized according to colony forming units (CFUs) or protein concentrations where indicated, resolved on SDS-PAGE and transferred onto Amersham Hybond-ECL nitrocellulose membranes (GE Healthcare). Western blots were probed using relevant primary antibodies, HRP-conjugated secondary anti-goat, mouse or rabbit antibodies, all diluted in in TBS-Tween 20 with 5% skim milk and developed using the ECL western blotting substrate (Thermo Scientific, Pierce Protein Biology Products). Signals were acquired using a Kodak Image Station 4000MM Pro and assembled for presentation using Adobe Photoshop CS3.

### Mice infection

Six to eight weeks old BALB/c female mice purchased from Jackson Labs (Bar Harbor, ME) were acclimated for a minimum of 1 week prior to infection and used in experimental groups of 5 animals. Mice were infected intraperitoneally (i.p.) with a total dose of approximately 10^5^ CFUs of *B. abortus* strain wild type strain 2308 or its isogenic *bsp* mutants suspended in 100 µl of PBS. Infectious doses were confirmed by plating serial dilutions of inocula on TSA plates. Groups of five mice per strain were euthanized at day 3, 7 and 42 post inoculation by isoflurane inhalation overdose followed by cervical dislocation. Spleens and livers were collected aseptically. Spleens were weighed to evaluate splenomegaly and bacteria were enumerated from livers and spleens through homogenization in PBS, plating of serial 10-fold dilutions on TSA plates and growth at 37°C for three days. All animal rearing, handling and experimental methods were conducted under protocols (#2010-043 and #2012-031) approved by the RML Institutional Animal Care and Use Committee (IACUC; USDA Permit Number: 51-F-0016, PHS number: A4149-01) in strict accordance with the recommendations of the Guide for the Care and Use of Laboratory Animals of the National Institutes of Health. All infections were performed in an Animal Biosafety Level 3 (ABSL3) facility according to protocols reviewed and approved by the Rocky Mountain Laboratories Institutional Biosafety Committee and the RML Institutional Animal Care and Use Committee (IACUC), in compliance with the CDC Division of Select Agents and Toxins regulations.

### Statistical analysis

Statistical analysis was performed using the GraphPad Prism software. All results are presented as means ± SD from at least three independent experiments, unless otherwise stated. Statistical significance was determined by either an unpaired, two-tailed Student *t* test or in the case of groups, a one-way ANOVA followed by the Tukey's test. A P value<0.05 indicates a statistically significant difference.

## Supporting Information

Figure S1 Representative fluorescence micrographs of J774A.1 cells infected with *B.* abortus expressing N-terminally tagged (TEM1-X) TEM1 fusion proteins and processed at 16 h post-infection for translocation of β-lactamase. (TIFF)

Figure S2 Representative fluorescence micrographs of J774A.1 cells infected with *B.* abortus expressing C-terminally tagged (X-TEM1) TEM1 fusion proteins and processed at 16 h post-infection for translocation of β-lactamase. (TIFF)

Figure S3 Intracellular growth and survival of *B. abortus* strain 2308 and its isogenic Δ*virB9* mutant strain within J774.A cells. (A) Cells were infected with either the wild-type 2308 or the Δ*virB9* mutant strains at an MOI of 1000 and intracellular CFUs enumerated at the indicated time points. (B) Similar to (A), J774.A1 cells were infected with either wild-type 2308 or the Δ*virB9* mutant strains at an MOI of 1000 and 5000, respectively, and intracellular CFUs enumerated at various time points. Arrow indicates the timepoint at which infected cells were processed for cAMP estimation. Data are means ± SEM from a representative experiment performed in triplicates. (TIF)

Figure S4 Assessment of ER stress in cells co-expressing HA- or GFP-tagged *Brucella* Bsp proteins and pSEAP-2 vector in HEK293T cells. (A) Expression of HA- or GFP-tagged *Brucella* Bsp proteins in HEK293T cells after 24 h of transfection. Samples resolved by SDS-PAGE were normalized to total protein concentrations. Asterisks indicate full-length proteins. (B) To monitor ER stress, HEK293T cells were co-transfected with ERSE reporter and indicated plasmids. After 32 h of transfection, Dual Luciferase assay was performed, and the promoter activity values calculated as described in the Materials and Methods. Cells expressing the ERSE reporter were also treated with the ER stress inducer Tunicamycin (5 µg/ml) for 8 h and assayed similarly. Data are means ± SD from a representative experiment performed three times in triplicates. (C) Western blot analysis of BiP and CHOP expression in HEK293T cells expressing HA- or GFP-tagged Bsp fusion proteins. Tunicamycin and HA-VceC were used as a positive control while single transfections with pSEAP-2 or co-transfections with empty vectors were used as negative controls. At 32 h post-transfection, cell lysates were generated and quantified using a BCA protein Assay Kit (Pierce). Equal amounts of samples were resolved by SDS-PAGE and immunoblotted with rabbit anti-BiP, mouse anti-CHOP and rabbit anti-Actin (as loading control) antibodies. (TIF)

Figure S5 Ectopic expression of BspA, BspB and BspF do not alter the morphology of the secretory compartment. HeLa cells expressing GFP-BspA, HA-BspB and HA-BspF were transfected for 12 h, fixed and immunostained using anti-HA, anti-COPII, anti-ERGIC-53 or anti GM130 antibodies and imaged by confocal microscopy. Images are representative micrographs. Insets in micrographs of GFP-BspA-expressing cells show accumulation of GFP-BspA in ERGIC structures (arrows) in addition to the ER. Scale bars, 10, 2 µm. (TIF)

Figure S6 Confirmation of in-frame deletion of *bsp* genes in *B. abortus* strain 2308. (A and B) Chromosomal loci flanking the deleted *bsp* genes were amplified by PCR using primers described in Table S3 and resolved by electrophoresis. (TIF)

Figure S7 *Brucella* infection does not affect the kinetics of Golgi re-assembly after BFA washout. HeLa cells either transfected with pSEAP-2 alone or infected with *B. abortus* strain 2308 or infected and transfected with pSEAP-2 were incubated with 5 µg/ml Brefeldin A (BFA) for 30 min, and then washed extensively. Cells were then fixed and immunostained for the Golgi apparatus using anti-GM130 antibodies, and changes in GM130 localization were monitored at the indicated times after BFA washout. Scale bar, 10 µm. (TIFF)

Figure S8 Representative expression of 3×FLAG-tagged Bsp proteins in *B. abortus* 2308. Whole cell lysates from strains carrying derivatives of either pJC123 or pJC124 expressing 3×FLAG-tagged BspA, BspB, BspF, BspD and BspE were resolved by SDS-PAGE and analyzed by Western blotting using either an anti-FLAG antibody to detect Bsp protein expression, or a anti-*Brucella* p17 protein to verify equal loading of samples. Asterisks indicate full length proteins. (TIF)

Figure S9 Effect of VirB T4SS deficiency on infection and persistence of *Brucella* in mice. Groups of five 6–8 weeks old BALB/c female mice were infected intraperitoneally (i.p.) with approximately 5×10^4^ CFUs of *B. abortus* strain 2308 and its isogenic Δ*virB9* mutant strain. On day 3, 7 and 42 mice were euthanized, and splenomegaly (A) and bacterial loads in spleens (B) and livers (C) were examined. (TIF)

Figure S10 Effect of single or combined deletions of *bspA*, *bspB* and *bspF* on infection and persistence of *Brucella* in mice. Groups of five 6–8 weeks old BALB/c female mice were infected intraperitoneally (i.p.) with approximately 1×10^5^ CFUs of *B. abortus* strain 2308 and its isogenic mutant strains. On day 3, 7 and 42 mice were euthanized, and splenomegaly (A and B) and bacterial loads in spleens (C and D) and livers (E and F) were examined. Asterisks indicate a statistically significant difference (P<0.05) as determined by a one-way ANOVA analysis followed by the Tukey's test. (TIF)

Table S1 List of predicted *B. abortus* proteins of unknown functions fulfilling search criteria for putative VirB T4SS effectors. (DOCX)

Table S2 List and features of *B. abortus* putative VirB T4SS effector proteins. (DOCX)

Table S3 Primers and plasmids used in this study. (DOCX)

Table S4 Primers used to construct and confirm *Brucella* in-frame deletion mutants. (DOCX)
