# Correction: *Brucella* modulates secretory trafficking via multiple Type IV secretion effector proteins

**DOI:** 10.1371/journal.ppat.1013302

**Published:** 2025-07-10

**Authors:** Sebenzile Myeni, Robert Child, Tony W. Ng, John J. Kupko III, Tara D. Wehrly, Stephen F. Porcella, Leigh A. Knodler, Jean Celli

Two gene locus numbers are incorrect due to typographical errors throughout the text:

bspE corresponds to locus tag number BAB1_1675, not BAB1_1671.

bspG corresponds to locus tag number BAB1_0277, not BAB1_0227.

In Figs 1 and 2, the two gene locus numbers are also incorrect. Please see the correct Figs 1 and 2 below.

**Fig 1:**
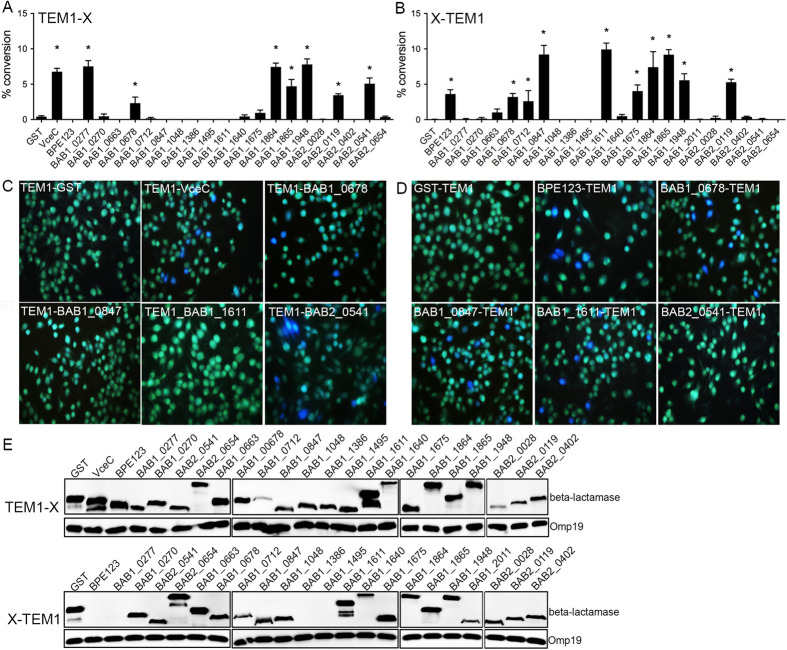
Translocation of *Brucella* putative effector proteins into J774A.1 cells. (A and B) Quantification of the translocation of N-terminally- (A) or C-terminally- (B) TEM1-tagged *Brucella* proteins. The cytosolic translocation of β-lactamase by *B. abortus* strain 2308 expressing the different TEM1 fusion proteins was assessed by fluorescence microscopy in J774.A1 macrophages after 16 h of infection. Cells from ten independent random fields were counted and the percentage of blue cells calculated. Results shown are the mean ± SD of three independent experiments. TEM1-GST and GST-TEM1 were used as negative controls (green fluorescence) and TEM1-VceC as a positive control (blue fluorescence). (C and D) Representative fluorescence micrographs from individual assay wells showing control proteins (GST and VceC) and selected TEM1 fusion proteins tagged at either the N-terminus (C) or C-terminus (D). (E) Western blot analysis showing expression of TEM-1 fusions. Bacterial lysates from *B. abortus* 2308 strain expressing TEM-1 fusion proteins were resolved using SDS-PAGE and subjected to Western blot analysis with anti- β-lactamase TEM-1 antibody. Detection of *Brucella* Omp19 was used as a loading control.

**Fig 2:**
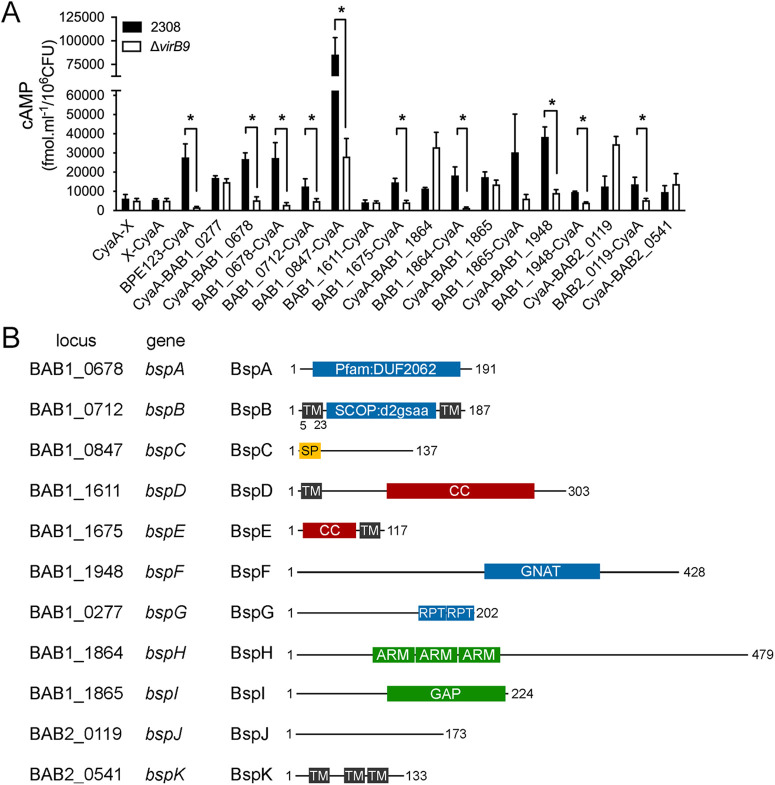
VirB-dependent translocation of *Brucella* Bsp effectors into J774. **A1 cells.** (A) *B. abortus* strain 2308 (black bars) or the Δ*virB9* mutant strain (white bars) expressing the CyaA fusions to the indicated *Brucella* proteins were used to infect J774.A1 cells, and the cAMP level of infected cells was determined. A previously identified VirB effector, BPE123, was used as a positive control while vectors expressing the CyaA domain alone (CyaA-X or X-CyaA) were used as a negative control. Total cAMP levels resulting from translocation of protein fusions were quantified and are expressed as fmol.ml^−1^/10^6^ CFUs. Results are means ± SD from a representative experiment performed in triplicate. (B) Schematic representation of *Brucella* secreted proteins (Bsp proteins), indicating the corresponding locus, protein lengths and predicted domains or motifs.
