# Supplementary figures and images for: Brucella Modulates Secretory Trafficking via Multiple Type IV Secretion Effector Proteins
Source: PLoS Pathog. 2013 Aug 8;9(8):e1003556. doi: 10.1371/journal.ppat.1003556 (PMC3738490; doi:10.1371/journal.ppat.1003556)

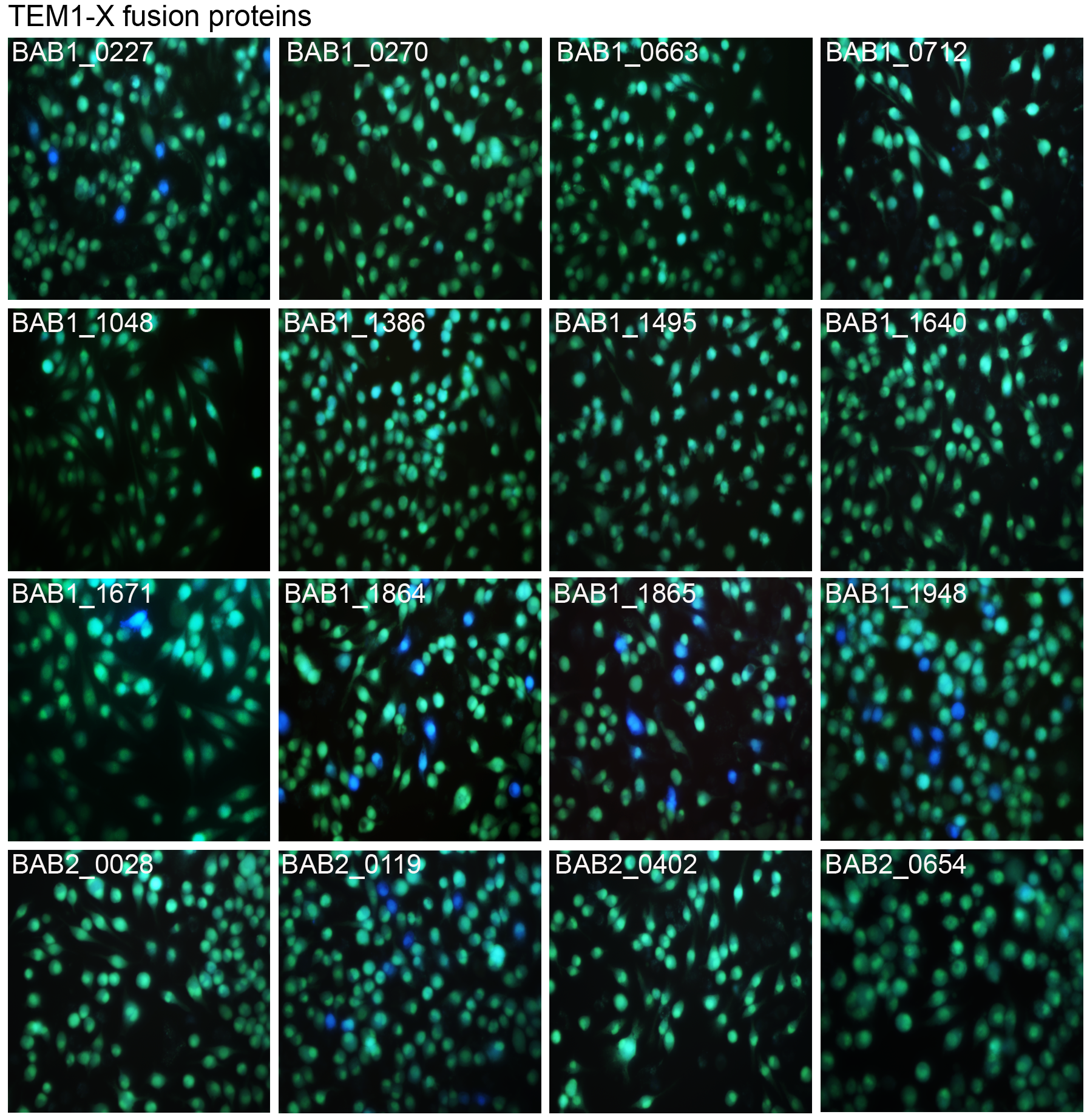

Supplement: Figure S1 — (TIFF) [file ppat.1003556.s001.tiff]

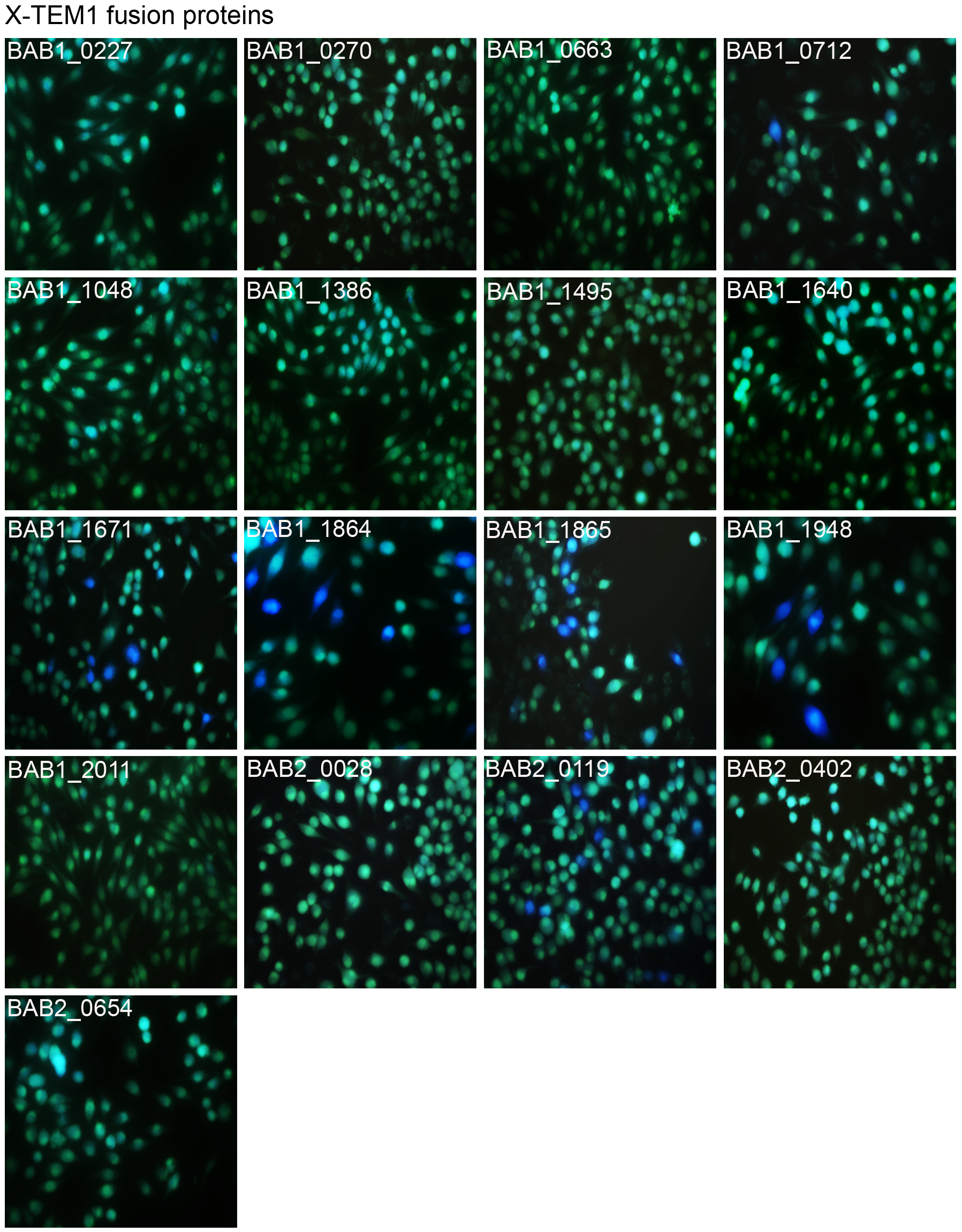

Supplement: Figure S2 — (TIFF) [file ppat.1003556.s002.tiff]

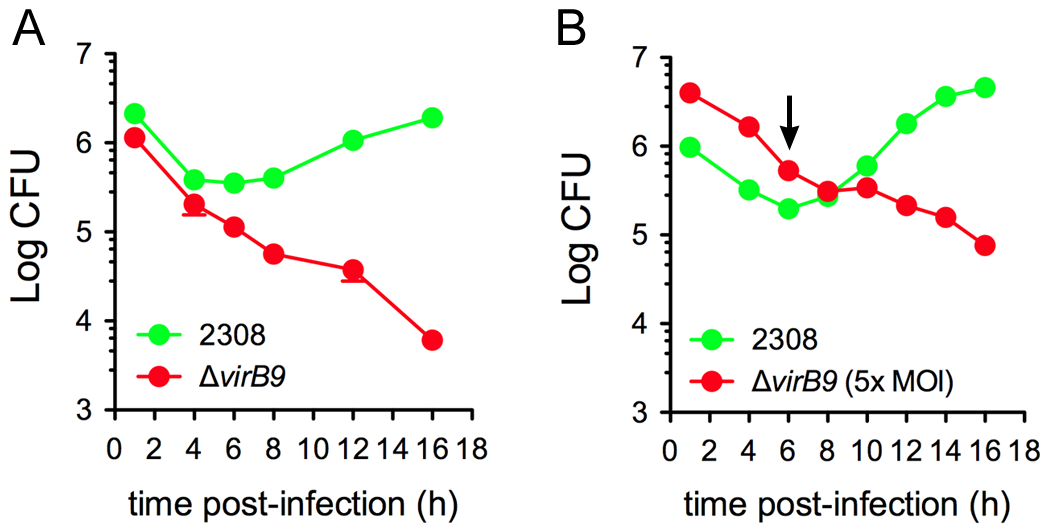

Supplement: Figure S3 — (TIF) [file ppat.1003556.s003.tif]

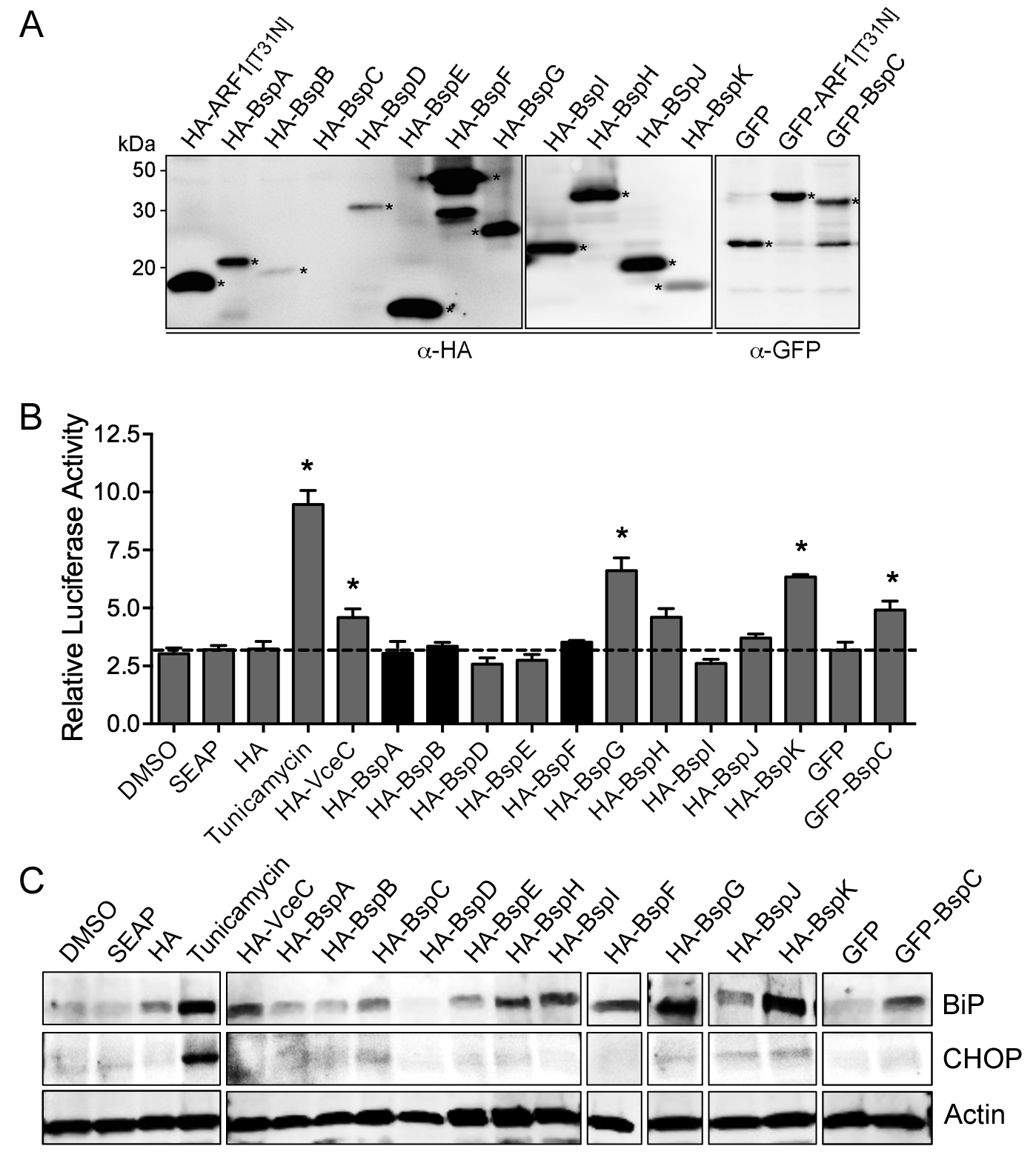

Supplement: Figure S4 — (TIF) [file ppat.1003556.s004.tif]

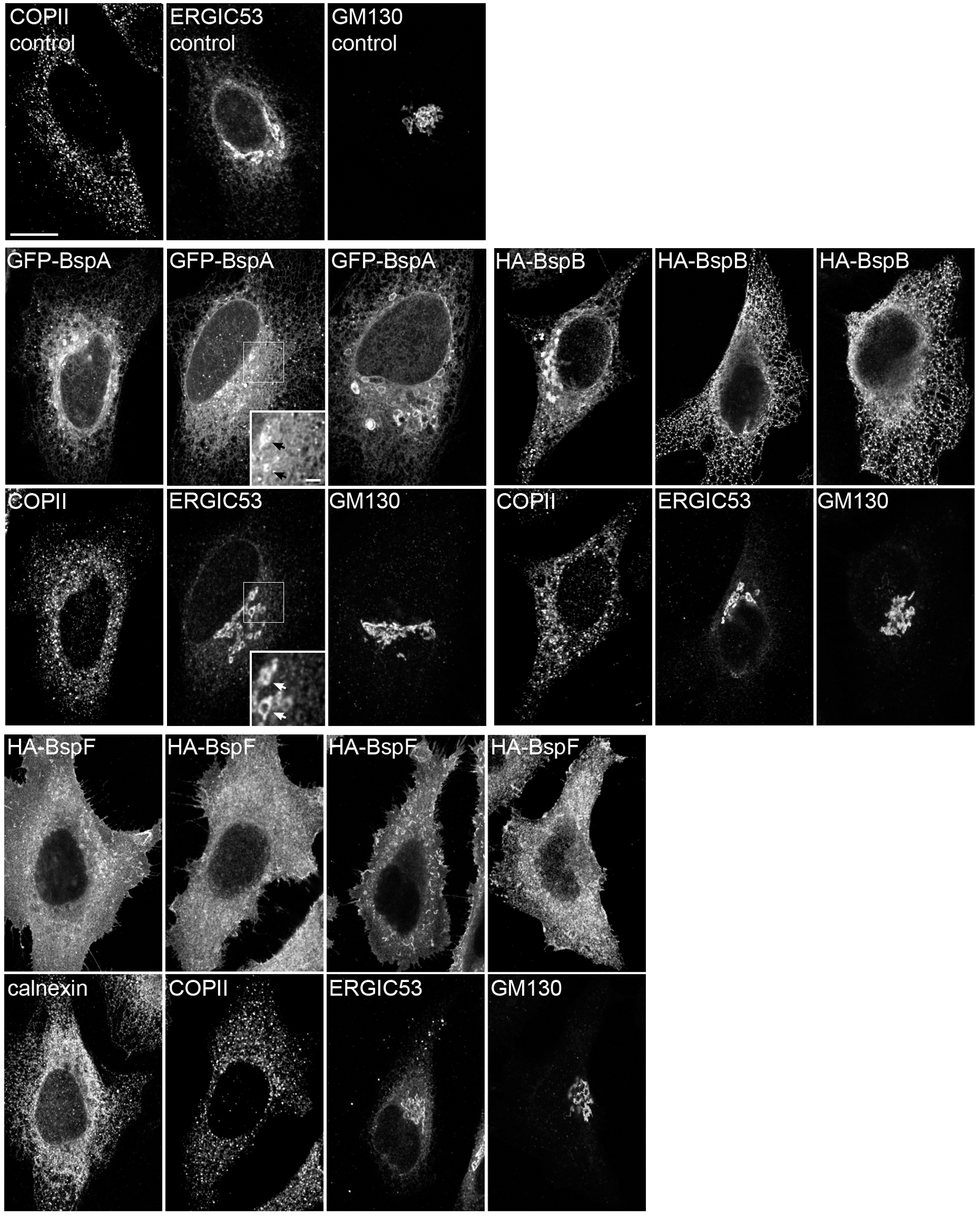

Supplement: Figure S5 — (TIF) [file ppat.1003556.s005.tif]

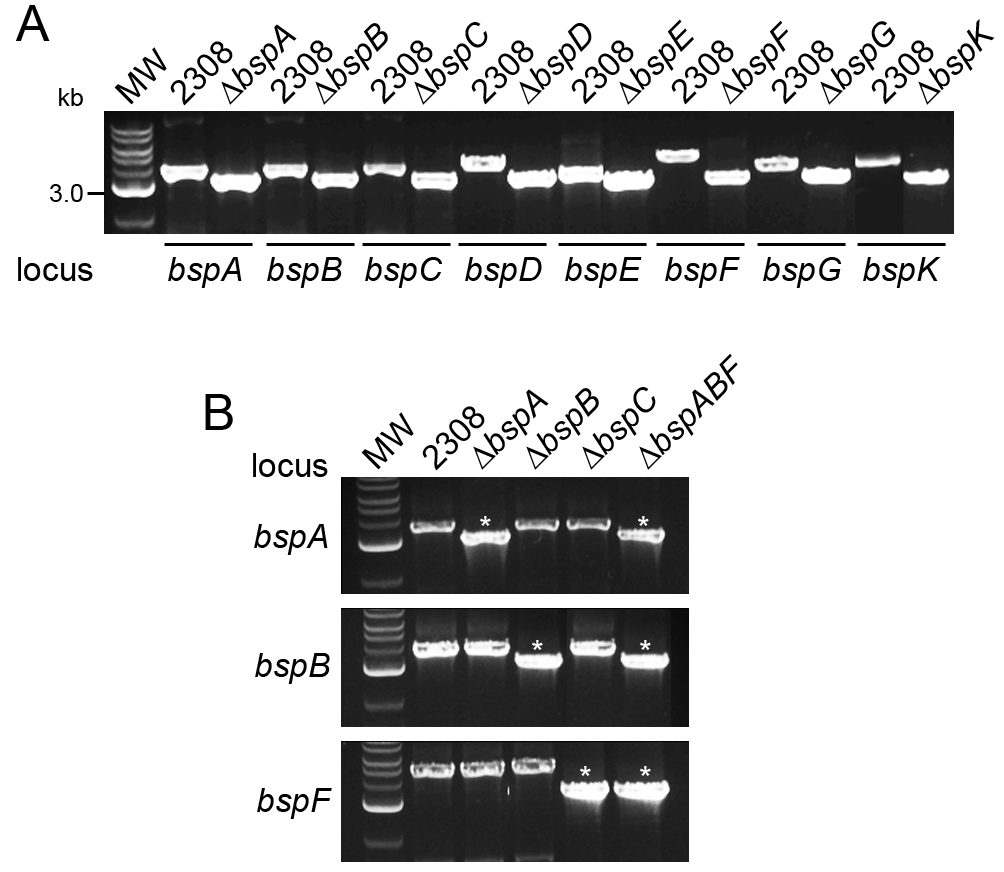

Supplement: Figure S6 — (TIF) [file ppat.1003556.s006.tif]

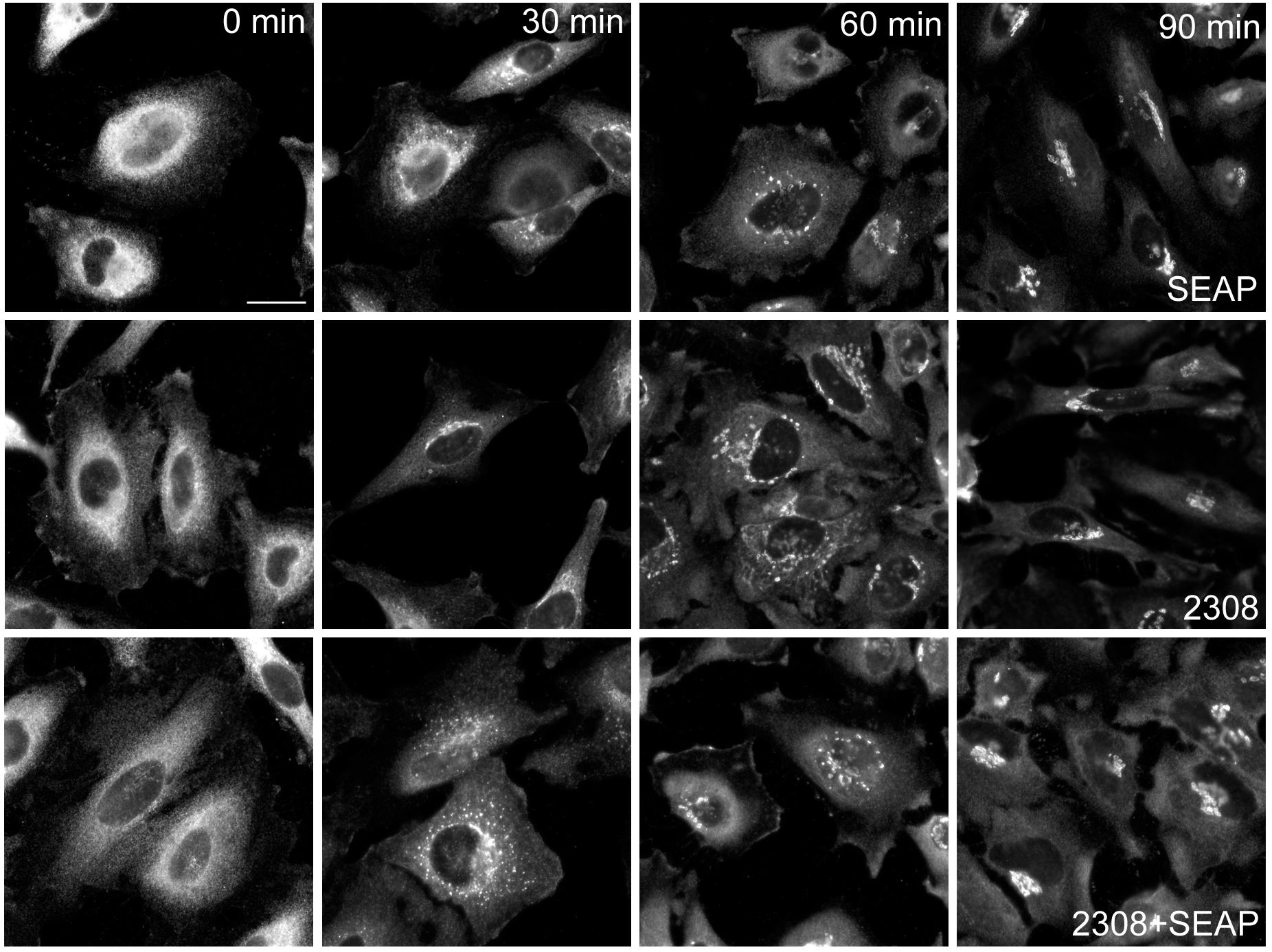

Supplement: Figure S7 — (TIFF) [file ppat.1003556.s007.tif]

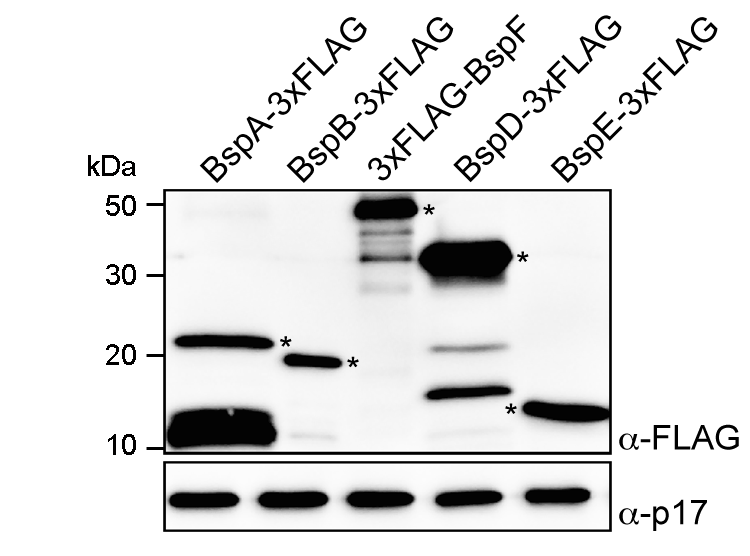

Supplement: Figure S8 — (TIF) [file ppat.1003556.s008.tif]

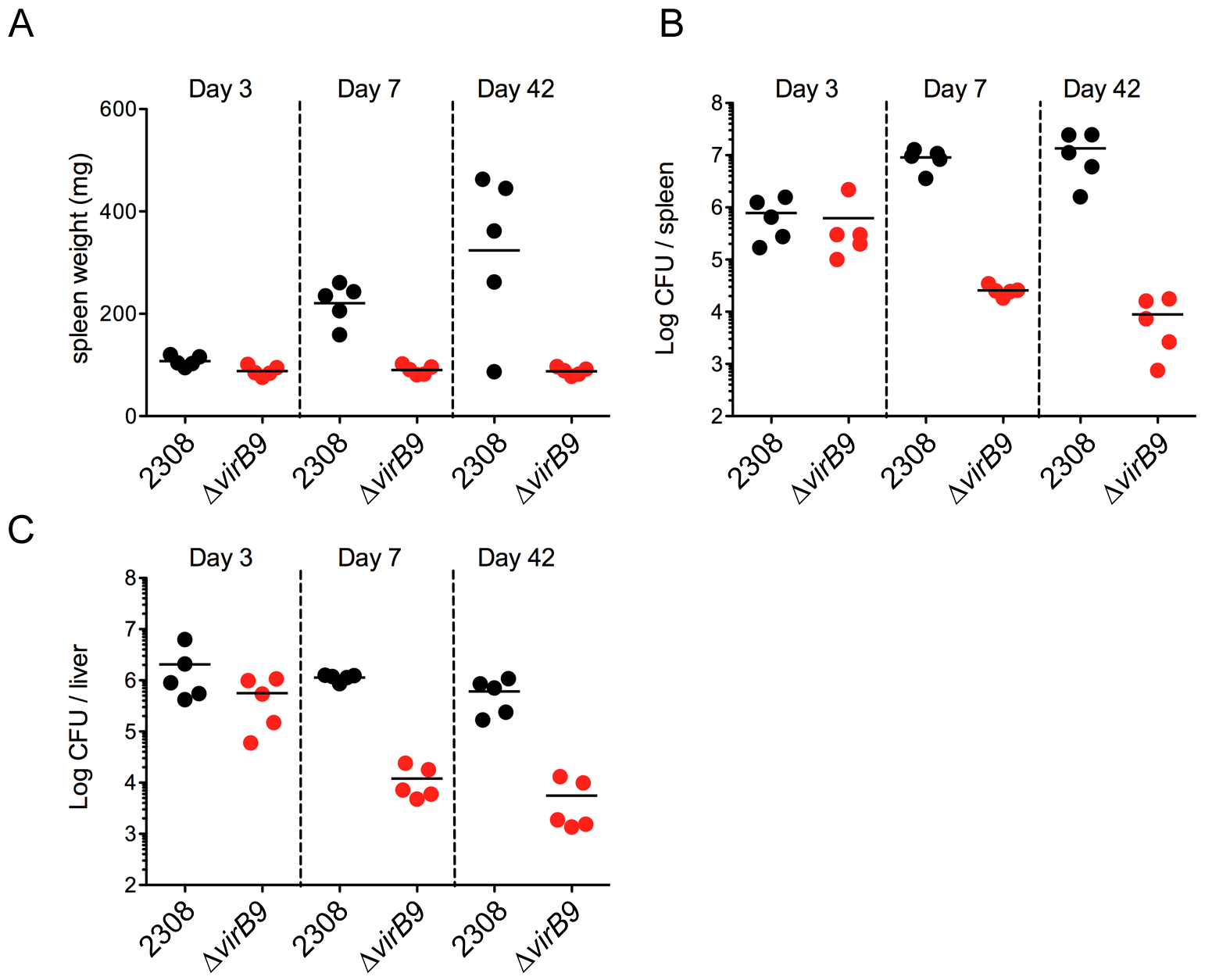

Supplement: Figure S9 — (TIF) [file ppat.1003556.s009.tif]

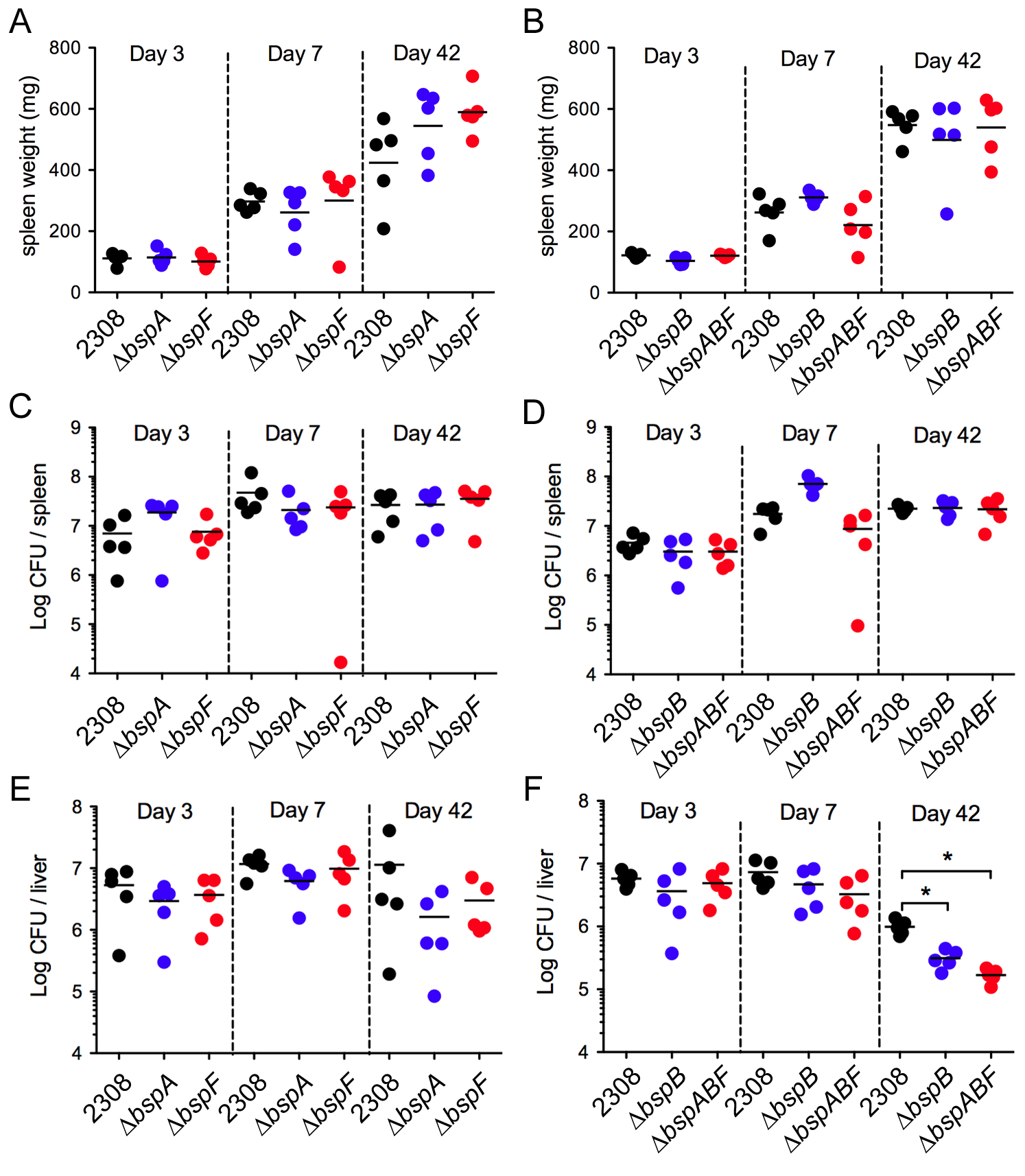

Supplement: Figure S10 — (TIF) [file ppat.1003556.s010.tif]
