# Supplementary material for: Brucella Modulates Secretory Trafficking via Multiple Type IV Secretion Effector Proteins
Source: PLoS Pathog. 2013 Aug 8;9(8):e1003556. doi: 10.1371/journal.ppat.1003556 (PMC3738490; doi:10.1371/journal.ppat.1003556)
Supplement: Table S1 — (DOCX) [file ppat.1003556.s011.docx]

Table S1: List of predicted *B. abortus* proteins of unknown functions fulfilling search criteria for putative VirB T4SS effectors

| locus^a^ | % Blastp hits within α2-proteobacteria^b^ | C-terminus 20 aa net charge^c^ | ∆GC content^d^ |
| --- | --- | --- | --- |
| BAB1_0592 | 100.0 | 6.98 | -1.23 |
| BAB1_0227 | 100.0 | 4.43 | -4.41 |
| BruAb1_0244 | 100.0 | 3.95 | -7.68 |
| BAB1_2011 | 100.0 | 2.95 | -10.97 |
| BAB2_0402 | 100.0 | 7.97 | -4.61 |
| BAB1_0013 | 100.0 | 5.22 | -21.01 |
| BAB1_1865 | 100.0 | 3.98 | -6.92 |
| BAB1_0847 | 100.0 | 3.95 | -5.15 |
| BAB1_0394 | 100.0 | 2.98 | -5.02 |
| BAB1_0123 | 100.0 | 2.98 | 7.27 |
| BAB2_0340 | 100.0 | 2.98 | -2.98 |
| BAB1_1492 | 100.0 | 2.97 | -0.03 |
| BAB1_1194 | 100.0 | 2.22 | 2.07 |
| BAB1_1835 | 100.0 | 2.22 | -2.48 |
| BAB2_0028 | 100.0 | 5.94 | -3.65 |
| BAB1_1485 | 100.0 | 3.19 | 1.84 |
| BAB1_0939 | 100.0 | 2.22 | 4.72 |
| BruAb1_1965 | 87.5 | 3.98 | 0.60 |
| BAB2_1030 | 83.3 | 4.22 | 0.41 |
| BAB2_0232 | 83.3 | 2.98 | 0.31 |
| BAB2_0450 | 80.0 | 3.98 | -0.49 |
| BAB2_0689 | 78.6 | 2.98 | 3.27 |
| BAB1_0750 | 75.0 | 3.22 | -9.56 |
| BAB2_0505 | 75.0 | 2.94 | 2.42 |
| BAB1_1048 | 71.4 | 4.22 | -0.59 |
| BAB1_1308 | 66.7 | 5.98 | 4.93 |
| BAB2_1162 | 66.7 | 4.98 | -5.73 |
| BAB2_0873 | 66.7 | 2.98 | -7.12 |
| BAB2_0473 | 66.7 | 2.19 | 1.45 |
| BAB1_0819 | 66.7 | 2.19 | -8.85 |
| BAB2_0676 | 66.7 | 4.22 | -11.27 |
| BAB1_1204 | 66.7 | 2.22 | 2.78 |
| BAB1_1615 | 66.7 | 2.98 | -0.30 |
| BAB1_0729 | 65.2 | 2.97 | 6.19 |
| BAB1_0438 | 64.7 | 3.22 | 5.01 |
| BAB1_1186 | 64.7 | 2.95 | -3.29 |
| BAB2_0195 | 63.6 | 3.98 | 5.26 |
| BAB1_1277 | 62.5 | 4.22 | 1.26 |
| BAB1_1457 | 61.5 | 3.95 | -0.53 |
| BAB1_1419 | 60.0 | 4.21 | 0.71 |
| BAB1_0519 | 55.6 | 4.98 | 3.92 |
| BAB1_1302 | 54.8 | 3.22 | 1.29 |
| BAB1_1795 | 54.5 | 2.98 | -2.54 |
| BAB2_0119 | 54.5 | 2.98 | -2.50 |
| BAB1_2017 | 54.5 | 2.98 | -2.12 |
| BAB1_0779 | 52.9 | 2.22 | -2.95 |
| BruAb1_0973 | 50.0 | 3.98 | -0.04 |
| BAB1_0712 | 50.0 | 3.98 | 5.17 |
| BAB1_1505 | 50.0 | 5.97 | -2.96 |
| BAB1_0782 | 50.0 | 3.98 | 1.34 |
| BAB1_0075 | 47.8 | 2.94 | 0.66 |
| BAB1_1912 | 45.5 | 3.22 | -1.46 |
| BAB1_1611 | 42.9 | 5.98 | -0.84 |
| BAB1_0915 | 42.9 | 3.22 | -0.43 |
| BAB1_1817 | 40.0 | 6.22 | -0.21 |
| BAB1_1426 | 40.0 | 3.22 | 1.73 |
| BAB2_0056 | 40.0 | 2.98 | 1.61 |
| BAB2_1067 | 37.5 | 2.19 | 0.86 |
| BAB1_1948 | 36.0 | 6.22 | 1.97 |
| BAB1_0709 | 35.7 | 2.98 | -1.17 |
| BAB1_0878 | 34.1 | 4.22 | 1.35 |
| BAB2_0541 | 33.3 | 4.67 | 1.68 |
| BAB2_0037 | 33.3 | 5.98 | 9.12 |
| BAB1_1043 | 33.3 | 2.22 | 2.45 |
| BAB1_0663 | 33.3 | 6.98 | 0.52 |
| BAB2_0959 | 30.0 | 4.22 | 1.55 |
| BAB1_1864 | 30.0 | 3.46 | -6.84 |
| BAB1_0329 | 29.3 | 4.19 | 4.26 |
| BAB1_0166 | 29.0 | 4.46 | 1.00 |
| BAB1_2007 | 27.8 | 2.22 | 4.86 |
| BAB1_1543 | 27.5 | 2.98 | -0.62 |
| BAB1_0655 | 27.0 | 2.94 | 0.80 |
| BAB1_0993 | 27.0 | 2.98 | -1.45 |
| BruAb1_1814 | 25.0 | 2.98 | 1.75 |
| BAB1_0735 | 25.0 | 2.46 | 5.49 |
| BAB1_0088 | 22.9 | 3.22 | 0.02 |
| BAB1_1386 | 22.2 | 3.22 | 5.44 |
| BAB2_0224 | 22.2 | 4.97 | 2.61 |
| BAB1_0678 | 22.0 | 4.46 | 4.04 |
| BAB2_0208 | 21.7 | 2.98 | 1.62 |
| BAB1_0740 | 20.0 | 2.98 | -1.10 |
| BAB1_1355 | 20.0 | 2.46 | -3.60 |
| BAB2_0057 | 20.0 | 2.98 | 0.53 |
| BruAb1_0022 | 20.0 | 3.46 | -3.52 |
| BAB1_1652 | 18.2 | 4.22 | -3.89 |
| BAB1_1640 | 18.2 | 3.98 | -3.67 |
| BAB2_0950 | 18.2 | 3.22 | 3.81 |
| BAB2_0339 | 15.8 | 4.46 | -1.19 |
| BAB2_0084 | 15.4 | 3.22 | 3.32 |
| BAB2_0880 | 14.6 | 3.22 | 0.99 |
| BAB1_0258 | 14.3 | 3.98 | -3.28 |
| BAB1_0270 | 13.6 | 4.98 | -8.32 |
| BAB2_0654 | 13.0 | 3.98 | 1.86 |

^a^ the BAB locus nomenclature refers to the *Brucella abortus* strain 2308 genome sequence; the BruAb locus nomenclature refers to the *Brucella abortus* strain 9-941 genome sequence. Loci listed with a BruAb number were not annotated in the strain 2308 genome sequence, although the corresponding open reading frames were present.

^b^ the number of Blastp hits within α2-proteobacteria, which was specific to *Brucella* spp. or not, was divided by the total number of hits and expressed as percentage.

^c^ the net charge of the C-terminal 20 aa residues was calculated using the EMBOSS tool “charge” (<http://emboss.bioinformatics.nl/>).

^d^ the ∆GC content is the deviation of the locus from the GC content of the entire genome (57.22%)
