# Supplementary material for: Brucella Modulates Secretory Trafficking via Multiple Type IV Secretion Effector Proteins
Source: PLoS Pathog. 2013 Aug 8;9(8):e1003556. doi: 10.1371/journal.ppat.1003556 (PMC3738490; doi:10.1371/journal.ppat.1003556)
Supplement: Table S2 — (DOCX) [file ppat.1003556.s012.docx]

Table S2: List and features of *B. abortus* putative VirB T4SS effector proteins

| locus^a^ | ∆GC content^b^ | C-terminal 20 aa | charge^c^ | features^d^ |
| --- | --- | --- | --- | --- |
| BAB1_0227 | -4.41 | AHQGWQKVPTKVGRKCHQLP | 4.43 | Internal repeat domains (RPT) |
| BAB1_0270 | -8.32 | GAADIRLNFMRKKWINKKGI | 4.98 | Metallopeptidase family |
| BAB1_0663 | +0.52 | TKKAANNVRSGTRRAKKQAG | 6.98 |  |
| BAB1_0678 | +4.04 | RLERLAEKHRLH**R**Q**R**ELQKA | 4.46 | *Pfam* DUF2062 |
| BAB1_0712 | +5.17 | VLGVLGLYGLMRT**R**L**R**RPQT | 3.98 | 2 TM; SCOP:d2gssa (PLP-dependent transferase) |
| BAB1_0847 | -5.15 | ITLRCGIT**R**G**K**LQAIEIVRK | 3.95 | Signal peptide (aa 1-22) |
| BAB1_1048 | -0.59 | KNRFERLLTYRLE**R**M**R**QTGH | 4.22 | Signal peptide (aa 1-23); CC domain |
| BAB1_1386 | +5.44 | LFLWAKSRGRASHSDRQREP | 3.22 | Signal peptide (aa 1-23); TM domain (aa 150-177) |
| BAB1_1495 | +1.84 | RHGRPTSVTFADTRGCPIIR | 3.19 | Signal peptide (aa 1-23) |
| BAB1_1611 | -0.84 | GKGKKNKAPKSLAT**R**I**R**DMQ | 5.98 | 1 TM (aa 5-27); CC domain (aa 86-273); |
| BAB1_1640 | -3.67 | AIRRIQFDAGVTKKQLLATK | 3.98 | Patatin-like phospholipase domain |
| BAB1_1671 | +0.61 | IASLATALGIGYLLALLSRR | ND | CC domain (aa 12-96); 1 TM domain; *Pfam* DUF883 |
| BAB1_1864 | -6.84 | IADLRKMHGRKHGFWPLVDR | 3.46 | Armadillo repeat (ARM) domains (aa 86-200) |
| BAB1_1865 | -6.92 | RQENMLDKLRKLVAFLG**R**F**R** | 3.98 | GTPase Activating Protein (GAP) domain |
| BAB1_1948 | +1.97 | RTRNLARII**R**N**R**L**R**SFTEHK | 6.22 | Acetyltransferase (GNAT) domain (aa 219-282) |
| BAB1_2011 | -10.97 | KSGYLRRSDMVPRFARDNCF | 2.95 | Signal peptide (aa 1-23) |
| BAB2_0028 | -3.65 | YRAVRKAKYSSLWIRSCYAK | 5.94 |  |
| BAB2_0119 | -2.50 | AEGFRATMLIRRDAVPRAYR | 2.98 |  |
| BAB2_0402 | -4.61 | AKQTYWYRVRKAVRRRLQGR | 7.97 | 1 TM (aa 76-98) |
| BAB2_0541 | +1.68 | YCAHNSHGFHAR**R**T**R**LSSGR | 4.67 | 3 TM domains |
| BAB2_0654 | +1.86 | AAALLDKMRSRAGLPPRPAR | 3.98 | VirJ homolog |

^a^ the locus nomenclature refers to the *Brucella abortus* strain 2308 genome sequence

^b^ the ∆GC content is the deviation of the locus from the GC content of the entire genome (57.22%)

^c^ the net charge of the C-terminal 20 aa residues was calculated using the EMBOSS tool “charge” (<http://emboss.bioinformatics.nl/>). ND, not determined

^d^ TM, transmembrane; CC, Coiled-coil; DUF, Domain of Unknown Function
