# Supplementary material for: Brucella Modulates Secretory Trafficking via Multiple Type IV Secretion Effector Proteins
Source: PLoS Pathog. 2013 Aug 8;9(8):e1003556. doi: 10.1371/journal.ppat.1003556 (PMC3738490; doi:10.1371/journal.ppat.1003556)
Supplement: Table S3 — (DOCX) [file ppat.1003556.s013.docx]

Table S3: Plasmids and primers used in this study

| plasmid | locus | forward primer | reverse primer |  |
| --- | --- | --- | --- | --- |
| pJC120-GST | - | **TW628**  TAAGCATTGGCTCGAGTCCCCTATACTAGGTTATTGG | **TW629**  CGACTCTAGACTCGAGTCAGTCACGATGCGGCCGCTC | |
| pJC120-BPE123 | BAB2_0123 | **TW422**  TAAGCATTGGCTCGAGAGCTTGTTGCTGGCTAACGTG | **TW423**  CGACTCTAGACTCGAGTCATGCCTGTCCCGCCAGTTC | |
| pJC120-BAB1_0227 | BAB1_0227 | **JC508**  GCGGTCGACAGCCAACCAGCCAATTCATCCG | **JC509**  GCGGAATTCTTAAGGTAATTGGTGGCATTTTCTGCCC | |
| pJC120-BAB1_0270 | BAB1_0270 | **TW470**  GCAGCCCGGGGGATCCAGCAGTCAGAATTACGTTGTC | **TW471**  TAGAACTAGTGGATCCTCAGATCCCTTTTTTATTGATC | |
| pJC120- BAB1_0663 | BAB1_0663 | **TW428**  TAAGCATTGGCTCGAGGAGTATGAGGACGAAATGC | **TW429**  CGACTCTAGACTCGAGTCAGCCAGCCTGTTTTTTTGC | |
| pJC120-BAB1_0678 | BAB1_0678 | **TW350**  TAAGCATTGGCTCGAGTTGTTCCAACGCAGATAT | **TW351**  CGACTCTAGACTCGAGTCATGCCTTCTGCAACTCCCG | |
| pJC120-BAB1_0712 | BAB1_0712 | **TW426**  TAAGCATTGGCTCGAGCGCCCCGTTCTTTTCCTGATG | **TW427**  CGACTCTAGACTCGAGTTATGTTTGGGGGCGGCGAAG | |
| pJC120-BAB1_0847 | BAB1_0847 | **TW434**  TAAGCATTGGCTCGAGAATCGACCAAGATCATACTTTCCGC | **TW435**  CGACTCTAGACTCGAGTTACTTGCGCACGATTTCTATG | |
| pJC120-BAB1_1048 | BAB1_1048 | **TW436**  TAAGCATTGGCTCGAGAATTTCAAGAAACGGGTTCTG | **TW437**  CGACTCTAGACTCGAGTCAATGGCCCGTCTGGCG | |
| pJC120-BAB1_1386 | BAB1_1386 | **TW430**  TAAGCATTGGCTCGAGAAAACCGCGCGCTTCCTGC | **TW431**  CGACTCTAGACTCGAGTCATGGCTCACGCTGCCG | |
| pJC120-BAB1_1495 | BAB1_1495 | **TW440**  TAAGCATTGGCTCGAGTCCGTTATTTCGCTCAAATCG | **TW441**  CGACTCTAGACTCGAGCTAGCGGATGATCGGGCAACCG | |
| pJC120-BAB1_1611 | BAB1_1611 | **TW424**  TAAGCATTGGCTCGAGATTCAATCCGCTCTCTTTTTG | **TW425**  CGACTCTAGACTCGAGCTATTGCATGTCGCGGATG | |
| pJC120-BAB1_1640 | BAB1_1640 | **JC504**  GCGGTCGACGGCCGGGCTATCTCACCAGTAACGG | **JC505**  GCGGAATTCCTACTTGGTTGCCAGCAATTGCTTC | |
| pJC120-BAB1_1671 | BAB1_1671 | **JC648**  GCGTCTAGAGCGCGTGCATCCGAGAAGCTG | **JC649**  GCGCTGCAGTCAGCGAATAGGGCGTTCACGG | |
| pJC120-BAB1_1864 | BAB1_1864 | **JC492**  GCGGTCGACGCATCAAAGACTACCTTGAATG | **JC493**  GCGGAATTCTCACCGATCTACAAGCGGCCAG | |
| pJC120-BAB1_1865 | BAB1_1865 | **JC494**  GCGGTCGACACTGACCTGATTCACATACATG | **JC495**  GCGGAATTCTCAGCGAAAGCGGCCCAAAAACG | |
| pJC120-BAB1_1948 | BAB1_1948 | **TW348**  TAAGCATTGGCTCGAGGCTGCAAAACCTTTGCTT | **TW349**  CGACTCTAGACTCGAGTTATTTATGCTCGGTGAAACT | |
| pJC120-BAB2_0028 | BAB2_0028 | **JC502**  GCGGTCGACGCGGCCCGGCCCATCGAAGCCC | **JC503**  GCGGAATTCTCACTTTGCGTAACAACTCCTG | |
| pJC120-BAB2_0119 | BAB2_0119 | **JC496**  GCGGTCGACCAGTTTTCAAAAGAGAGAGAAACC | **JC497**  GCGGAATTCTTATCGATATGCCCGAGGTACAGC | |
| pJC120-BAB2_0402 | BAB2_0402 | **JC488**  GCGGAGCTCCTCAAAGGTCCAGCCTTTAGCC | **JC489**  GCGGAATTCTCAGCGGCCTTGCAGTCGCCGCC | |
| pJC120-BAB2_0541 | BAB2_0541 | **TW352**  TAAGCATTGGCTCGAGGTGGCGGGTCTTTCAGTGGTA | **TW353**  CGACTCTAGACTCGAGTCACCGTCCTGAAGAAAGCCG | |
| pJC120-BAB2_0654 | BAB2_0654 | **TW432**  TAAGCATTGGCTCGAGAAGAAAGAACGCGTATTC | **TW433**  CGACTCTAGACTCGAGTCAGCGCGCAGGGCGCGGC | |
| pJC121-GST | - | **TW630**  AATGCTCGAGGTCGACTCCCCTATACTAGGTTATTGG | **TW631**  TATCGATACCGTCGACGTCACGATGCGGCCGCTC | |
| pJC121-BPE123 | BAB2_0123 | **TW368**  AATGCTCGAGGTCGACAGCTTGTTGCTGGCTAACGTG | **TW369**  TATCGATACCGTCGACTGCCTGTCCCGCCAGTTCAAC | |
| pJC121-BAB1_0227 | BAB1_0227 | **TW382**  AATGCTCGAGGTCGACAGCCAACCAGCCAATTCATCC | **TW383**  TATCGATACCGTCGACAGGTAATTGGTGGCATTTTCT | |
| pJC121-BAB1_0270 | BAB1_0270 | **TW388**  AATGCTCGAGGTCGACAGCAGTCAGAATTACGTTGTC | **TW389**  TATCGATACCGTCGACGATCCCTTTTTTATTGATCCA | |
| pJC121-BAB1_0663 | BAB1_0663 | **TW396**  AATGCTCGAGGTCGACGAGTATGAGGACGAAATGC | **TW397**  TATCGATACCGTCGACGCCAGCCTGTTTTTTTGCG | |
| pJC121-BAB1_0678 | BAB1_0678 | **TW402**  AATGCTCGAGGTCGACTTGTTCCAACGCAGATATCCT | **TW403**  TATCGATACCGTCGACTGCCTTCTGCAACTCCCGCTG | |
| pJC121-BAB1_0712 | BAB1_0712 | **TW384**  AATGCTCGAGGTCGACCGCCCCGTTCTTTTCCTGATG | **TW385**  TATCGATACCGTCGACTGTTTGGGGGCGGCGAAGGCG | |
| pJC121-BAB1_0847 | BAB1_0847 | **TW406**  AATGCTCGAGGTCGACAAATCGACCAAGATCATACTTTC | **TW407**  TATCGATACCGTCGACCTTGCGCACGATTTCTATGGC | |
| pJC121-BAB1_1048 | BAB1_1048 | **TW410**  AATGCTCGAGGTCGACAATTTCAAGAAACGGGTTCTGGC | **TW411**  TATCGATACCGTCGACATGGCCCGTCTGGCGCATCCG | |
| pJC121-BAB1_1386 | BAB1_1386 | **TW398**  AATGCTCGAGGTCGACAAAACCGCGCGCTTCCTGCTG | **TW399**  TATCGATACCGTCGACTGGCTCACGCTGCCGGTCTGA | |
| pJC121-BAB1_1495 | BAB1_1495 | **TW412**  AATGCTCGAGGTCGACTCCGTTATTTCGCTCAAATCG | **TW413**  TATCGATACCGTCGACGCGGATGATCGGGCAAC | |
| pJC121-BAB1_1611 | BAB1_1611 | **TW370**  AATGCTCGAGGTCGACATTCAATCCGCTCTCTTT | **TW371**  TATCGATACCGTCGACTTGCATGTCGCGGATGCG | |
| pJC121-BAB1_1640 | BAB1_1640 | **TW386**  AATGCTCGAGGTCGACGGCCGGGCTATCTCACC | **TW387**  TATCGATACCGTCGACCTTGGTTGCCAGCAATT | |
| pJC121-BAB1_1671 | BAB1_1671 | **TW392**  AATGCTCGAGGTCGACGCGCGTGCATCCGAGAA | **TW393**  TATCGATACCGTCGACGCGGCGCGAGAGCAAGG | |
| pJC121-BAB1_1864 | BAB1_1864 | **TW372**  AATGCTCGAGGTCGACGCATCAAAGACTACCTTGAAT | **TW373**  TATCGATACCGTCGACCCGATCTACAAGCGGCCAGAA | |
| pJC121-BAB1_1865 | BAB1_1865 | **TW374**  AATGCTCGAGGTCGACACTGACCTGATTCACATACA | **TW375**  TATCGATACCGTCGACGCGAAAGCGGCCCAAAAACG | |
| pJC121-BAB1_1948 | BAB1_1948 | **TW394**  AATGCTCGAGGTCGACGCTGCAAAACCTTTGCTTGAG | **TW395**  TATCGATACCGTCGACTTTATGCTCGGTGAAACTGCG | |
| pJC121-BAB1_2011 | BAB1_2011 | **TW408**  AATGCTCGAGGTCGACCAATGGTGTTTTTCTGC | **TW409**  TATCGATACCGTCGACAAAACAATTATCACGTGCG | |
| pJC121-BAB2_0028 | BAB2_0028 | **TW376**  AATGCTCGAGGTCGACGCGGCCCGGCCCATCGAAGC | **TW377**  TATCGATACCGTCGACCTTTGCGTAACAACTCCTGA | |
| pJC121-BAB2_0119 | BAB2_0119 | **TW378**  AATGCTCGAGGTCGACCAGTTTTCAAAAGAGAGAGAA | **TW379**  TATCGATACCGTCGACTCGATATGCCCGAGGTACAGC | |
| pJC121-BAB2_0402 | BAB2_0402 | **TW380**  AATGCTCGAGGTCGACCTCAAAGGTCCAGCCTTT | **TW381**  TATCGATACCGTCGACGCGGCCTTGCAGTCGCCG | |
| pJC121-BAB2_0541 | BAB2_0541 | **TW404**  AATGCTCGAGGTCGACGTGGCGGGTCTTTCAGTGGTACG | **TW405**  TATCGATACCGTCGACCCGTCCTGAAGAAAGCCGTGTGC | |
| pJC121-BAB2_0654 | BAB2_0654 | **TW400**  AATGCTCGAGGTCGACAAGAAAGAACGCGTATTC | **TW401**  TATCGATACCGTCGACGCGCGCAGGGCGCGGCG | |
| pJC125-BAB1_0227 | BAB1_0227 | **RC102**  GCTTGATATCGAATTCAGCCAACCAGCCAATTCATC | **RC103**  CGGGCTGCAGGAATTCTTAAGGTAATTGGTGGCAT | |
| pJC125-BAB1_0678 | BAB1_0678 | **RC41**  GCTTGATATCGAATTCTTGTTCCAACGCAGATATCCTC | **RC42**  CGGGCTGCAGGAATTCTCATGCCTTCTGCAACTCCCGC | |
| pJC125-BAB1_1864 | BAB1_1864 | **TW686**  GCTTGATATCGAATTCGCATCAAAGACTACCTTG | **TW687**  CGGGCTGCAGGAATTCTCACCGATCTACAAGCG | |
| pJC125-BAB1_1865 | BAB1_1865 | **TW690**  GCTTGATATCGAATTCACTGACCTGATTCACATACATG | **TW691**  CGGGCTGCAGGAATTCTCAGCGAAAGCGGCCCAAAAAC | |
| pJC125-BAB1_1948 | BAB1_1948 | **RC37**  GCTTGATATCGAATTCGCTGCAAAACCTTTGCTTGAG | **RC38**  CGGGCTGCAGGAATTCTTATTTATGCTCGGTGAAACT | |
| pJC125-BAB2_0119 | BAB2_0119 | **RC25**  GCTTGATATCGAATTCCAGTTTTCAAAAGAGAGAG | **RC26**  CGGGCTGCAGGAATTCTTATCGATATGCCCGAGGTAC | |
| pJC125-BAB2_0541 | BAB2_0541 | **RC45**  GCTTGATATCGAATTCGTGGCGGGTCTTTCAGTGGTAC | **RC46**  CGGGCTGCAGGAATTCTCACCGTCCTGAAGAAAGCCG | |
| pJC126-BAB1_0678 | BAB1_0678 | **RC43**  AATGCTCGAGGTCGACTTGTTCCAACGCAGATATCCT | **RC44**  TATCGATACCGTCGACTGCCTTCTGCAACTCCCGCTG | |
| pJC126-BAB1_0712 | BAB1_0712 | **RC31**  AATGCTCGAGGTCGACCGCCCCGTTCTTTTCCTGATG | **RC32**  TATCGATACCGTCGACTGTTTGGGGGCGGCGAAGGCG | |
| pJC126-BAB1_0847 | BAB1_0847 | **RC51**  AATGCTCGAGGTCGACAAATCGACCAAGATCATACTTTC | **RC52**  TATCGATACCGTCGACCTTGCGCACGATTTCTATGGC | |
| pJC126-BAB1_1611 | BAB1_1611 | **TW700**  AATGCTCGAGGTCGACATTCAATCCGCTCTCTTT | **TW701**  TATCGATACCGTCGACTTGCATGTCGCGGATGCG | |
| pJC126-BAB1_1671 | BAB1_1671 | **RC35**  AATGCTCGAGGTCGACGCGCGTGCATCCGAGAA | **RC36**  TATCGATACCGTCGACGCGGCGCGAGAGCAAGG | |
| pJC126-BAB1_1864 | BAB1_1864 | **TW688**  AATGCTCGAGGTCGACGCATCAAAGACTACCTTGAAT | **TW689**  TATCGATACCGTCGACCCGATCTACAAGCGGCCAGAA | |
| pJC126-BAB1_1865 | BAB1_1865 | **TW692**  AATGCTCGAGGTCGACACTGACCTGATTCACATACA | **TW693**  TATCGATACCGTCGACGCGAAAGCGGCCCAAAAACG | |
| pJC126-BAB1_1948 | BAB1_1948 | **RC39**  AATGCTCGAGGTCGACGCTGCAAAACCTTTGCTTGAG | **RC40**  TATCGATACCGTCGACTTTATGCTCGGTGAAACTGCG | |
| pJC126-BPE123 | BAB2_0123  (BPE123) | **TW696**  AATGCTCGAGGTCGACAGCTTGTTGCTGGCTAACGTG | **TW697**  TATCGATACCGTCGACTGCCTGTCCCGCCAGTTCAAC | |
| pJC126-BAB2_0119 | BAB2_0119 | **RC27**  AATGCTCGAGGTCGACCAGTTTTCAAAAGAGAGAGAA | **RC28**  TATCGATACCGTCGACTCGATATGCCCGAGGTACAGC | |
| pCMV-HA-*vceC* | BAB1_1058 (*vceC*) | **JC907**  AGC**GAATTC**AAATGAAGGAATGGCTCAGCGG | **JC908**  ATTCTCGAGCTAATTGCGGGTTTCTCCCT | |
| pCMV-HA-*bspA* | BAB1_0678 | **TW758**  ATGGAGGCCCGAATTCAATTGT  TCCAACGCAGATATCC | **TW759**  TCGGTCGACCGAATTCTCATG  CCTTCTGCAACTCCC | |
| pCMV-HA-*bspB* | BAB1_0712 | **TW752**  ATGGAGGCCCGAATTCAACGCCCCGTTCTTTTCCTGAT | **TW753**  TCGGTCGACCGAATTCTTATGTTTGGGGGCGGCGAA | |
| pCMV-HA-*bspC* | BAB1_0847 | **TW762**  ATGGAGGCCCGAATTCAAAAATCGACCAAGATCATACTTTCCGC | **TW763**  TCGGTCGACCGAATTCTTACTTGCGCACGATTTCTA | |
| pCMV-HA-*bspD* | BAB1_1611 | **TW710**  ATGGAGGCCCGAATTCAAATTCAATCCGCTCTCTTT | **TW711**  TCGGTCGACCGAATTCCTATTGCATGTCGCGGATGCG | |
| pCMV-HA-*bspE* | BAB1_1671 | **TW754**  ATGGAGGCCCGAATTCAAGCGCGTGCATCCGAGAAGCT | **TW755**  TCGGTCGACCGAATTCTCAGCGGCGCGAGAGCAA | |
| pCMV-HA-*bspF* | BAB1_1948 | **TW756**  ATGGAGGCCCGAATTCAAGCTGCAAAACCTTTGCTTGA | **TW757**  TCGGTCGACCGAATTCTTATTTATGCTCGGTGAAACTGCG | |
| pCMV-HA-*bspG* | BAB1_0227 | **TW716**  ATGGAGGCCCGAATTCAAAGCCAACCAGCCAATTCATC | **TW717**  TCGGTCGACCGAATTCTTAAGGTAATTGGTGGCATTTTCTGC | |
| pCMV-HA-*bspH* | BAB1_1864 | **TW708**  ATGGAGGCCCGAATTCAAGCATCAAAGACTACCTTGAAT | **TW709**  TCGGTCGACCGAATTCTCACCGATCTACAAGCGGCCAGAA | |
| pCMV-HA-*bspI* | BAB1_1865 | **TW706**  ATGGAGGCCCGAATTCAAACTGACCTGATTCACATACATG | **TW707**  TCGGTCGACCGAATTCTCAGCGAAAGCGGCCCAAAAAC | |
| pCMV-HA-*bspJ* | BAB2_0119 | **TW750**  ATGGAGGCCCGAATTCAACAGTTTTCAAAAGAGAGAG | **TW751**  TCGGTCGACCGAATTCTTATCGATATGCCCGAGGTAC | |
| pCMV-HA-*bspK* | BAB2_0541 | **TW760**  ATGGAGGCCCGAATTCAAGTGGCGGGTCTTTCAGTGGT | **TW761**  TCGGTCGACCGAATTCTCACCGTCCTGAAGAAAGCC | |
| pEGFP-N1-*bspA* | BAB1_0678 | **TW786**  TCAAGCTTCGAATTCTGCAG**GCCACC**ATGTTGTTCCAACGCAGATA | **TW787**  CGCGGTACCGTCGACTGCAGTGCCTTCTGCAACTCCCGCT | |
| pEGFP-N1-*bspC* | BAB1_0847 | **TW790**  TCAAGCTTCGAATTCTGCAG**GCCACC**GTGATTCAATCCGCTCTCTT | **TW791**  CGCGGTACCGTCGACTGCAGTTGCATGTCGCGGATGCG | |
| pEGFP-C1-*bspD* | BAB1_1611 | **TW722**  GTCCGGACTCAGATCTATTCAATCCGCTCTCTTT | **TW723**  CTTGAGCTCGAGATCTCTATTGCATGTCGCGGATGCG | |
| pmTn*7*K | - | **RC7**  GAGCTCATGCATGATATCGCGAACCGGAATTGCCAGC | **RC8**  TGCGGCCCGGACGATATCTCAGAAGAACTCGTCAAGA | |
| pmTn*7*K-*bspB* | BAB1_0712 | **RC389**  ATGCGCCCCGTTCTTTTCCTG  **RC360**  GCATGAGCTCACTAGTGGATTGCTTCGTCACCGATTT | **RC390**  GCAGCCCGGGGGATCCTTATGTTTGGGGGCGGCGAAG  **RC388**  AAGAACGGGGCGCATGATAACTCCAGACTTGGATT | |
| pmTn*7*K-*bspF* | BAB1_1948 | **RC395**  GCATGAGCTCACTAGTACCATCTTCCGATCTTGGC | **RC396**  GCAGCCCGGGGGATCCTTATTTATGCTCGGTGAAACTGCG | |
| pJC123-BspA | BAB1_0678 | **TW484**  GCAGCCCGGGGGATCCTTGTTCCAACGCAGATATCCTC | **TW485**  TAGAACTAGTGGATCCTCATGCCTTCTGCAACTCCCGC | |
| pJC123-BspB | BAB1_0712 | **TW466**  GCAGCCCGGGGGATCCCGCCCCGTTCTTTTCCTGATG | **TW467**  TAGAACTAGTGGATCCTTATGTTTGGGGGCGGCGAAG | |
| pJC124-BspF | BAB1_1948 | **TW358**  ATGATAAAGAATTCCTGCAGGCTGCAAAACCTTTGCTTGAG | **TW359**  GTGGATCCCCCGGGCTGCAGTTATTTATGCTCGGTGAAACT | |
| pJC123-BspD | BAB1_1611 | **TW452**  GCAGCCCGGGGGATCCATTCAATCCGCTCTCTTTTTG | **TW453**  TAGAACTAGTGGATCCCTATTGCATGTCGCGGAT | |
| pJC123-BspE | BAB1_1671 | **TW474**  GCAGCCCGGGGGATCCGCGCGTGCATCCGAGAAGC | **TW475**  TAGAACTAGTGGATCCTCAGCGGCGCGAGAGCAAG | |
|  |  |  |  | |
