# Supplementary material for: Brucella Modulates Secretory Trafficking via Multiple Type IV Secretion Effector Proteins
Source: PLoS Pathog. 2013 Aug 8;9(8):e1003556. doi: 10.1371/journal.ppat.1003556 (PMC3738490; doi:10.1371/journal.ppat.1003556)
Supplement: Table S4 — (DOCX) [file ppat.1003556.s014.docx]

Table S4: Primers used to construct and confirm *Brucella* in-frame deletion mutants

| Primer name | Sequence (5' - 3') | Primer function |
| --- | --- | --- |
| Δ*bspA* | | |
| TW840 | CGGTACCCGGGGATCCCCAGCAGCAGGAATGAATAATG | 1480 bp upstream, start codon, and first two codons of *bspA* |
| TW841 | GAACAACATGCGCTTCTAATA | 1480 bp upstream, start codon, and first two codons of *bspA* |
| TW842 | AAGCGCATGTTGTTCGCATGATCGTCGGAATTGGAA | Last codon of *bspA*, stop codon, and 1373 bp downstream |
| TW843 | ATGCCTGCAGGTCGACTTCCAGCCGTTTCAGATTG | Last codon of *bspA*, stop codon, and 1373 bp downstream |
| TW848 | CATCGGCTTGCGGTCGGTAT | ∆*bspA* |
| TW849 | CACCGCTGACCCAGGTAAAC | ∆*bspA* |
| TW850 | CACCTGGAACAGCGATTGCG | ∆*bspA* within the BAB1_0678 locus |
| TW851 | ACAATTCGCCTTCCGATGCT | ∆*bspA* within the BAB1_0678 locus |
| Δ*bspB* | | |
| TW804 | CGGTACCCGGGGATCCTCATTCAGGCACGCAACCTC | 1410 bp upstream and start codon of *bspB* |
| TW805 | CATCAGGCTTTACGCTCCC | 1410 bp upstream and start codon of *bspB* |
| TW806 | GTAAAGCCTGATGCAAACATAAGAGCCTGTTCC | Last two codons of *bspB,* stop codon, and 1391 bp downstream |
| TW807 | ATGCCTGCAGGTCGACATCTCGACCTTGAAGGCGTC | Last two codons of *bspB,* stop codon, and 1391 bp downstream |
| TW812 | TTGAAGAAGCTCGGCATGG | ∆*bspB* |
| TW813 | CTCGACCAGAAGAAGATCAG | ∆*bspB* |
| TW814 | AACGGCGAGCCCAAACATGC | ∆*bspB* within the BAB1_0712 locus |
| TW815 | GCCGAACCCGATCTTCTTCT | ∆*bspB* within the BAB1_0712 locus |
| Δ*bspC* | | |
| TW864 | CGGTACCCGGGGATCCGCGATCTTCATCCACGGGAC | 1478 bp upstream, start codon, and first three codons of *bspC* |
| TW865 | GGTCGATTTCATGCCCCGTTT | 1478 bp upstream, start codon, and first three codons of *bspC* |
| TW866 | GGGCATGAAATCGACCCGCAAGTAACCGTTTTCTTGC | Last two codons of *bspC*, stop codon, and 1427 bp downstream |
| TW867 | ATGCCTGCAGGTCGACCGTTTCTTACCAGACGGCT | Last two codons of *bspC*, stop codon, and 1427 bp downstream |
| TW872 | CGTCTTGTCAAGTTCACGGG | ∆*bspC* |
| TW873 | CGAACCGTCTAGGCATTGAG | ∆*bspC* |
| TW874 | GCTTGCTCATCGCATTTCCT | ∆*bspC* within the BAB1_0847 locus |
| TW875 | TGGATGGTGCGGTTGAGCGTG | ∆*bspC* within the BAB1_0847 locus |
| Δ*bspD* | | |
| TW900 | CGGTACCCGGGGATCCCACCTTCTTCCAGCAATTCC | 1359 bp upstream, start codon, and first five codons of *bspD* |
| TW901 | GAGAGCGGATTGAATCACGATTTC | 1359 bp upstream, start codon, and first five codons of *bspD* |
| TW902 | GATTCAATCCGCTCTCATGCAATAGAGCATTTTCGC | Last two codons of *bspD*, stop codon, and 1356 bp downstream |
| TW903 | ATGCCTGCAGGTCGACCGCTTCGTAATCCAGCACAT | Last two codons of *bspD*, stop codon, and 1356 bp downstream |
| TW908 | CACCAAGATCGAGAGCTGA | ∆*bspD* |
| TW909 | CACGCCAACGACGTCATAAC | ∆*bspD* |
| TW910 | GGAGTTTCACCTTGTTCTTG | ∆*bspD* within the BAB1_1611 locus |
| TW911 | CAAACTTGACGGTCTGGTTG | ∆*bspD* within the BAB1_1611 locus |

| Δ*bspE* | | |
| --- | --- | --- |
| TW816 | CGGTACCCGGGGATCCTGATGAAGCAGATGCGGTTAC | 1392 bp upstream, start codon, and first codon of *bspE* |
| TW817 | CGCCATGTCTGGTCTCCTGTT | 1392 bp upstream, start codon, and first codon of *bspE* |
| TW818 | AGACCAGACATGGCGCGCTGAGCCGCACCATGCTGAA | Last codon of *bspE*, stop codon, and 1419 bp downstream |
| TW819 | ATGCCTGCAGGTCGACGGAACGCAATGAACGCATCA | Last codon of *bspE*, stop codon, and 1419 bp downstream |
| TW824 | CAACGACGATCCCCTGCCT | ∆*bspE* |
| TW825 | TGGGGCTGCTTCTCTTTACC | ∆*bspE* |
| TW826 | CAGACCTGATATGCCTTATTG | ∆*bspE* within the BAB1_1671 locus |
| TW827 | CAAGCTGATCCATGAGAACC | ∆*bspE* within the BAB1_1671 locus |
| Δ*bspF* | | |
| TW828 | CGGTACCCGGGGATCCCTGTCGTTGCAGGACGATCT | 1481 bp upstream, start codon, and first two codons of *bspF* |
| TW829 | TGCAGCCATTACAACCTCTCC | 1481 bp upstream, start codon, and first two codons of *bspF* |
| TW830 | GTTGTAATGGCTGCAAAATAACGGGCAAATTAACCG | Last codon of *bspF,* stop codon, and 1596 bp downstream |
| TW831 | ATGCCTGCAGGTCGACGAATATGGGTCTGTTCGTTG | Last codon of *bspF,* stop codon, and 1596 bp downstream |
| TW836 | GCAACTTTGAAATCCGCAAG | ∆*bspF* |
| TW837 | ACCTCGGACAAGATGTGATC | ∆*bspF* |
| TW838 | ACGGAACGCCATGAAAGCC | ∆*bspF* within the BAB1_1948 locus |
| TW839 | GAAGAGATTGTCGTGCGAG | ∆*bspF* within the BAB1_1948 locus |
| Δ*bspG* | | |
| TW888 | CGGTACCCGGGGATCCGTATCATAATCGGTGCGAAG | 1420 bp upstream, start codon, and first 3 codons of *bspG* |
| TW889 | TGGTTGGCTCATAGTCCTTGC | 1420 bp upstream, start codon, and first 3 codons of *bspG* |
| TW890 | ACTATGAGCCAACCACCTTAAAGGAATACCTTACAG | Last codon of *bspG*, stop codon, and 1411 bp downstream |
| TW891 | ATGCCTGCAGGTCGACCCAACAAGCTATCAGTTCAG | Last codon of *bspG*, stop codon, and 1411 bp downstream |
| TW896 | GTCGTACAGCAAACGCCCGA | ∆*bspG* |
| TW897 | GCAAAATGTCAGAGCTTGAG | ∆*bspG* |
| TW898 | AAGGTCTGGATATTGACGTTC | ∆*bspG* within the BAB1_0227 locus |
| TW899 | CACCATGAGTTCGTACTCTTC | ∆*bspG* within the BAB1_0227 locus |
| Δ*bspK* | | |
| TW852 | CGGTACCCGGGGATCCGATATGACAGCCCGCGACAGT | 1484 bp upstream, start codon, and first codon of *bspK* |
| TW853 | CACCATGGAGAGGCCTTTCTG | 1484 bp upstream, start codon, and first codon of *bspK* |
| TW854 | GGCCTCTCCATGGTGGGACGGTGATCAACATGTTTG | Last two codons of *bspK*, stop codon, and 1343 bp downstream |
| TW855 | ATGCCTGCAGGTCGACGCTTGTCTGACTAACTCTCT | Last two codons of *bspK*, stop codon, and 1343 bp downstream |
| TW860 | GTTTCTCACGCAGTCGGCAAG | ∆*bspK* |
| TW861 | GTGCGACGAGGGTGAGATTG | ∆*bspK* |
| TW862 | CCTATATCATTGCCTATGCC | ∆*bspK* within the BAB2_0541 locus |
| TW863 | CAAGGAGCATCACGACTTCTTC | ∆*bspK* within the BAB2_0541 locus |

|  |
| --- |
